# Employable as We Age? A Systematic Review of Relationships Between Age Conceptualizations and Employability

**DOI:** 10.3389/fpsyg.2020.605684

**Published:** 2021-02-05

**Authors:** Annet H. De Lange, Beatrice Van der Heijden, Tinka Van Vuuren, Trude Furunes, Christiane De Lange, Josje Dikkers

**Affiliations:** ^1^Department of Human Resource Management, HAN University of Applied Sciences, Nijmegen, Netherlands; ^2^Department of Psychology, Universidade da Coruna, A Coruña, Spain; ^3^Faculty of Psychology, Open University Heerlen, Heerlen, Netherlands; ^4^Norwegian School of Hotel Management, University of Stavanger, Stavanger, Norway; ^5^Faculty of Psychology, Norwegian University of Science and Technology, Trondheim, Norway; ^6^Institute for Management Research, Radboud University, Nijmegen, Netherlands; ^7^School of Management, Open University of the Netherlands, Heerlen, Netherlands; ^8^Department of Marketing, Innovation and Organisation, Ghent University, Ghent, Belgium; ^9^Hubei Business School, Hubei University, Wuhan, China; ^10^Kingston Business School, Kingston University, London, United Kingdom; ^11^a.s.r. Loyalis, Heerlen, Netherlands; ^12^University of Stavanger, Stavanger, Norway; ^13^Open University of the Netherlands, Heerlen, Netherlands; ^14^HAN University of Applied Sciences, Nijmegen, Netherlands; ^15^HU University of Applied Sciences Utrecht, Utrecht, Netherlands

**Keywords:** employability, systematic review, functional age, organizational age, age conceptualizations

## Abstract

This systematic review aimed to provide an overview of earlier research on the relationships between age conceptualizations (i.e., calendar age, organizational age, lifespan age, psychosocial age, and functional age) and indicators of employability. We have conducted a systematic literature search using PsycINFO, Academic Search Premier, Business Source Complete, CINAHL, ERIC, MEDLINE, and Science Direct. Two raters evaluated the articles and subsequently distinguished *k* = 41 studies that met the inclusion criteria for this systematic review. Our review revealed that many researchers adopted different operationalizations to measure employability (15 studies were based on an input- or competence-based measure of employability, 23 studies included an output- or labor market-based measure of employability, and three studies included a combination of both measures). Moreover, most studies included calendar age (40 studies, 97.6%) as indicator of aging at work, and were based on a cross-sectional design (34 studies, 82.9%; 17.1% a longitudinal design). Based on the Standardized Index of Convergence (SIC) method, different types of evidence were found for the relationships between age and the employability measures. For relationships between psychosocial age and lifespan age, on the one hand, and employability measures, on the other hand, too few studies were found to draw conclusions. Yet, for relationships between calendar age and labor market-based measures strong consistent negative relationships were found across the studies, and moderately strong positive relationships were found for functional age and labor market- based measures. For organizational age and both competence-based as well as labor market-based measures moderately strong negative relationships were found. We discuss the implications of these results and propose a research agenda for future studies.

In the dynamic labor market, mainly through robotization and technological innovation, workers have to learn to effectively self-manage, develop, or foster their employability (i.e., career potential (Van der Heijde and Van der Heijden, 2006) into old age (Berntson et al., 2008; De Lange et al., 2015; Kooij, 2015) in order to protect and preferably even further enhance their career sustainability (Van der Heijden et al., 2020). The notion of sustainable careers has urged the need to gain more insight into the evolution of workers' employability across the lifespan.

The interest into the topic of employability is reflected in the fact that over the years the concept is studied across various disciplines (e.g., labor economics, education, careers, human resource management) focusing on different perspectives (Thijssen et al., 2008). Inspired by the sustainable career paradigm (De Vos and Van der Heijden, 2015), this particular contribution is meant to respond to one of the key challenges for societies nowadays, that is the aging of the workforce population, herewith stressing the importance to better understand how aging might potentially impact one's employability throughout the course of one's working life.

Besides changes in career-related requirements, workers also have to cope with or compensate for so-called age-related losses in abilities as well as changing opportunities at work (i.e., reduced physical reserves as one ages, changing cognitive abilities, and a changing social position; based on a literature review of De Lange et al., 2020) that can affect their chances for employment now and/or in the future. These developments refer to losses as well as gains in relation to work functioning [in terms of abilities, motivation, and opportunities at work; see the AMO-framework by Appelbaum et al. (2000)], and are either more intra-personal (e.g., changes in physical reserves) or more interpersonal in nature (e.g., social perceptions or stereotypes). As a result, aging is a complex process that may affect the employability levels of aging workers in different ways.

Earlier research has suggested that lifespan changes, like decreasing mental as well as physical reserves, can significantly affect the reported levels of employability (Brooke and Taylor, 2005; Ng and Feldman, 2009; Vandenberghe et al., 2012; Nilsson and Ekberg, 2013; Rusanova, 2014; Bal and De Lange, 2015; De Lange et al., 2015; Kooij et al., 2015), but no study to date has actually presented an overview of previous empirical work on the relationships between age conceptualizations and employability to draw more firm conclusions. Moreover, given the fact that previous scholarly efforts on aging at work and employability are limited and conceptually diverse (see also Kooij et al., 2008; Dikkers et al., 2017), we argue that it is important to incorporate multiple age conceptualizations, besides only calendar age (see also Le Blanc et al., 2017; Zacher et al., 2018).

In particular, as calendar age is considered a proxy measure for many complex age-related changes (Kooij, 2015; Zacher et al., 2018), and given that people become more heterogeneous with age (Staudinger and Bowen, 2011), we posit that a broader conceptualization of aging at work is needed in order to add to our scholarly knowledge in this field. To further understand the nature and direction of possible relationships between aging and employability at work, this study aimed to present a synthesis of existing published research in peer-reviewed journals in this scholarly domain. Before addressing the specific questions that are central to this review, we will start with a discussion of the theoretical background and definitions of the concepts of employability as well as aging at work.

## Employability: Definitions, Conceptualizations, and Theory

Throughout Western societies, we perceive a move away from a paternalistic toward a performance culture, and from providing a lifetime employment in one's working organization to requiring sustainable employability and adaptability across different work settings. The valuable historical overview presented by Versloot et al. (1998) clarifies the changes in focus of attention regarding the phenomenon of employability over the past decades. For example, in the early nineties, employers would often invest in the employability of their personnel in order to make it possible for them to find other work (also referred as outplacement). In this way, they endeavored to prevent forced dismissal (Van der Heijden et al., 2020).

In more recent decades, the concept of employability has been conceptualized, either based on a so-called competence-based (antecedents or inputs of employability) or as a labor market-based view of employability (employability as output variable). Within the competence-based approach of employability, scholars look at knowledge, skills, and attitudes, or more general competencies to assess employability (e.g., Fugate et al., 2004; Van der Heijde and Van der Heijden, 2006), whereas the labor market-based approach of employability focuses on individuals' perceptions of being able to obtain and retain a job, and into labor market positions or transitions available between positions as indicators of employability (Vanhercke et al., 2014; Veld et al., 2015; Nelissen, 2016).

Given this diversity in employability research, many theoretical underpinnings are cited (see Elsey, 2016 for an elaborate overview), depending upon the proposed perspective that is taken to the concept. For instance, building upon Conservation of Resources (COR) theory (Hobfoll, 2001), employability is defined as personal resources which enable individuals to better cope with challenging situations (De Cuyper et al., 2012), and can promote well-being and career success (Vanhercke et al., 2014), while, based upon Social Exchange Theory (SET) (Blau, 1964), employability is interpreted to be the responsibility of an individual as well as their organization (Veld et al., 2015).

Originally, the concept of employability was mainly operationalized by self-reported vs. supervisor-rated type of scales (Vanhercke et al., 2014; Guilbert et al., 2015). For example, self-reported perceived employability can be defined as the individual's perception of available possibilities of obtaining and maintaining employment (adapted from Berntson and Marklund, 2007; Vanhercke et al., 2014). More specifically, following the competence-based perspective of employability, we can distinguish specific skills and talents-based definitions (e.g., learned skills at work; Van der Heijde and Van der Heijden, 2006), while disposition-based definitions refer to individual differences (for example, attitudes related to career and work in general; Fugate and Kinicki, 2008). These two different approaches of employability are combined in a process model of the development of employability across time (Forrier et al., 2009; Vanhercke et al., 2014). In this process model, employability is the result of a complex integration of personal factors, structural or contextual factors, and their interactions over time, all of which affect options for employment. In this context, personal factors are tied to the person (e.g., age, gender differences, competences, dispositions etc.), whereas structural or contextual factors can play a role at the level of the job (e.g., work characteristics: Forrier and Sels, 2003; Griffeth et al., 2005; Vanhercke et al., 2014), the organization (e.g., support for career development, possible age-sensitive culture; Ng et al., 2005), or the society (e.g., total number of available jobs: Forrier and Sels, 2003; McQuaid and Lindsay, 2005; Rothwell and Arnold, 2007).

For example, older workers perceive fewer chances for employment compared to younger workers in the labor market (Forrier et al., 2009). One reason is that older workers are perceived to be more expensive compared to their younger counterparts because their wages are likely to increase with their organizational tenure. To further address the possible relationships between the multiple indicators of aging at work and indicators of employability, we first need to address the available conceptualizations of the concept of Aging at work.

## Conceptualizations of Aging at Work

*Aging at work* can best be operationalized as a multi-dimensional process including changes in psychological, physical, social, as well as societal functioning across time (De Lange et al., 2006; Kooij et al., 2008; Zacher et al., 2018). Sterns and Doverspike (1989) proposed five different approaches; chronological, functional, psychosocial, organizational, and lifespan development, to measure these changes across time.

*Chronological* age refers to one's calendar age. As chronological age increases, individuals go through various biological and psychological changes that are reflected in individuals' health, cognitive abilities, psychical capacity, and performance. Calendar age is by far the most widely used age conceptualization, and previous empirical work has found negative relationships with perceived labor market opportunities (e.g., Van der Heijden et al., 2009; De Cuyper et al., 2011; Van Vuuren et al., 2011), which can be attributed to age-related stereotyping and discrimination which is reflected in less appreciation of older workers and diminished efforts to invest in their future career development (Truxillo et al., 2015).

*Functional* or performance-based age is based on a worker's performance, and can cover a great variation in abilities and functioning across different age groups as well as within age groups (Sharkey, 1987). Van Vuuren and Marcelissen (2017) found in their study on functional age, measured as work ability, a positive association with employability.

*Psychosocial* or subjective age is based on the self-perception or social perception of age. Subjective age (or self-perception) refers to how old an individual feels, looks, and acts, the age cohort with which the individual identifies, and how old the person desires to be (Kaliterna et al., 2002; Stephan et al., 2012). For example, workers who feel young have a more open subjective time perspective whereas workers who feel old appear to have a more closed time-till-retirement perspective, which affects their career-related goals and self-regulation strategies (De Lange et al., 2011; Kanfer et al., 2013).

*Organizational* age refers to the aging of individuals in jobs and organizations, which is in the literature often described in terms of seniority, and job or organizational tenure. When people start their careers, their person-vocation fit is often quite low (Lang and Carstensen, 2002; De Lange et al., 2010; Kooij et al., 2010; Kanfer et al., 2013). Over the years, through trial and error learning, most employees discover jobs that suit them best, and gain more insight into which competencies need further development. In a later career stage, the problem of “experience concentration” or skill obsolescence (Thijssen, 1992) might occur, due to the fact that people have been specializing themselves so strongly, and over such a long period of time. More specifically, by performing the same job for many years, it becomes difficult for them to find or to learn another job (Van der Heijden and Thijssen, 2003). If their job becomes superfluous (e.g., the job of a mechanic at a telecommunications company) or if their health suffers from doing the same job for decades (e.g., the emotionally and physically demanding job of firemen), their employability is seriously at risk (Nauta et al., 2005). Conversely, Kooij et al. (2008) found that age-related factors can have a positive effect as well, reflected in the rise in salary with aging.

The *lifespan* concept of age relates to behavioral changes at any point in the life cycle and goes into the intra-individual changes associated with individuals moving through (older) adulthood. Lifespan age can be measured, for example, by life stage or family status (Sterns and Doverspike, 1989; De Lange et al., 2006). We can describe lifespan as a sequence of positions a person holds over a period of time (e.g., Kanfer et al., 2013). Substantial events – such as getting a new job, getting married, having children, experiencing loss of a loved one – mark significant transitions from one position or “social identity” to another one that affects career-related attitudes, choices and strategies, and subsequently one's reported level of employability. For example, compared to older workers, in general, work appears to be less central in the lives of 25–44 year-old workers, and this applies especially to female part-time workers (Warr, 2008). For employees with children, it is highly important to be able to combine work and private life flexibly; therefore, it is obvious that lifespan age affects one's employability chances.

Lifespan age is also related to the financial possibility to retire early (De Wind et al., 2014). In a similar vein, one's partner's labor market situation, measured in terms of wishes and increased value placed on leisure time, may influence retirement decisions (Van Dam et al., 2009; Syse et al., 2014), and their motivation to (not) continue working and/or retire as well (Kooij et al., 2008). Finally, McQuaid and Lindsay (2005) found that individual household circumstances (i.e., caring responsibilities, financial, emotional, and/or time commitments to relatives) are also related to workers' employability.

De Lange et al. (2006) translated the above-explained five different types of operationalizations into an integrative figure, highlighting the relationships between general aging at work and possible underlying age-related factors and their accompanying operationalizations (see also **Figure 2**). Elaborating on this, Kooij et al. (2008) conducted a review of 24 empirical and nine conceptual studies on the aforementioned age operationalizations (including different age measures) in relation to work motivation, and found that most of the distinguished age-related factors have a negative effect on the motivation of aging people to continue to work (Dikkers et al., 2017). However, Kooij et al. (2008) did not include indicators of employability in their review.

*Theorizing on successful aging*. During the last decades, *lifespan theories* have developed from unilateral perceptions of age toward more complex, multi-dimensional, or dynamic conceptualizations of the aging process (De Lange et al., 2015; Dikkers et al., 2017, Kooij, 2015). For example, both the lifespan theory of Selection Optimization and Compensation (SOC) (Baltes et al., 1999) and the Socio-emotional Selectivity Theory (Carstensen et al., 1999; Carstensen, 2006) highlight the process of conservation of personal as well as career-related resources through self-regulatory compensatory goal-related choices and coping strategies. In particular, the *Selection Optimization and Compensation theory (SOC)* (Baltes et al., 1999; Kooij, 2015; Dikkers et al., 2017; Zacher et al., 2018) hypothesizes that people maximize gains and minimize losses they experience over time using different strategies. To maximize gains, people select or choose desirable outcomes or goals (i.e., elective selection) and optimize their resources and environment to reach these desired career or personal life goals and outcomes. To minimize losses, people select fewer goals in response to these losses and compensate for them by investing their remaining resources. By employing these strategies, individuals strive to achieve three lifespan goals, namely: growth (i.e., reaching higher levels of functioning); maintenance (i.e., maintaining or returning to current levels of functioning); and regulation of loss (i.e., functioning adequately at lower levels). SOC theory proposes that the allocation of resources used for maintenance and regulation of loss will increase with age, whereas resources aimed at growth will decrease with age (Baltes et al., 1999).

The *Lifespan Theory of Control* (Heckhausen et al., 2010) builds upon the SOC theory by addressing how individuals pro-actively choose goals in accordance with the principles of developmental optimization (Dikkers et al., 2017). The theory proposes that aging workers who experience more losses (i.e., physical reserves) across time will learn to develop a greater reliance on secondary control strategies, i.e., strategies that address internal motivational processes to minimize losses of primary control over important outcomes in one's environment. Secondary control strategies are needed when the original goal has become unattainable. With aging, people must increasingly rely on such secondary control strategies to keep striving for the maximization of primary control. Secondary control strategies can also help minimize further losses and maintain current levels of functioning or expand primary control. For example, an individual whose functional capacities decrease with age could change his/her mastery goals from a competitive performance orientation to a mastery-avoidance goal, and focus on one's own behavior and preventing further loss in work-related functioning across time (e.g., by selecting work that fits their strengths or experience at work; De Lange et al., 2010; Kanfer et al., 2013).

Similar to the aforementioned lifespan developmental theories, theorizing and empirical research in the field of careers also speculates that employability is the result of career goals and regulation strategies to maximize the career-related outcomes (Van der Heijden et al., 2020). For example, Gottfredson (2002) *career circumscription and compromise theory* defined career compromise as changing occupational preferences under pressing external circumstances, and argued that career choice results from two processes. First, individuals circumscribe or eliminate unacceptable occupational options based on factors such as age, gender, prestige level, and interest. Moreover, compromise refers to the process by which their most preferred options are modified or relinquished across time. Second, occupational goals that have been identified as desirable might still be eliminated or modified if the individual determines that they will be unattainable (active coping strategies). Furthermore, circumscription is the process whereby individuals reject career alternatives due to, for example, reduced physical health.

In sum, the aforementioned theories suggest that aging workers will report more losses, in terms of physical resources (De Lange et al., 2020) and negative age perceptions or discrimination at work (Guilbert et al., 2018), and therefore may experience a diminishing output- or labor market- based employability across time. As a result, our review may find more negative relationships between age conceptualizations, on the one hand, and output- or labor market-based employability, on the other hand. Furthermore, earlier research also reveals differences in terms of loss and growth among aging workers in terms of competencies across the life course (De Vos et al., 2017; Van der Heijden et al., 2020); suggesting mixed relationships between age conceptualizations and input-based or competence-based employability.

Despite the previous scholarly work regarding theorizing and research concerning the concepts of aging at work and employability, an overview of empirical research examining their relationships is still missing. We argue that such a review would allow us to draw more firm conclusions regarding the nature and direction of possible relationships between age conceptualizations and indicators of employability. Therefore, we have formulated the following review questions as guidelines underlying our scholarly work:

- What are relevant descriptives of the reviewed studies (in terms of type of research population, and country)?;
- What are the operationalizations of the concept of age used in these empirical studies (e.g., chronological, functional, psychosocial, organizational, lifespan age)?;
- What are the operationalizations of the concept of employability used in these studies (e.g., competence-based vs. labor market-based measures or both types of measures included)?;
- Which research designs were used in these reviewed studies (e.g., cross-sectional or longitudinal survey research)?;
- What is the direction and the strength of evidence of the relationships between the different age operationalizations and employability in these studies (e.g., significant positive, negative or non-significant effects based on the SIC method; see Method)?

The responses to the aforementioned questions will help us to develop a new research agenda for future studies.

## Methods

**Figure 1:**
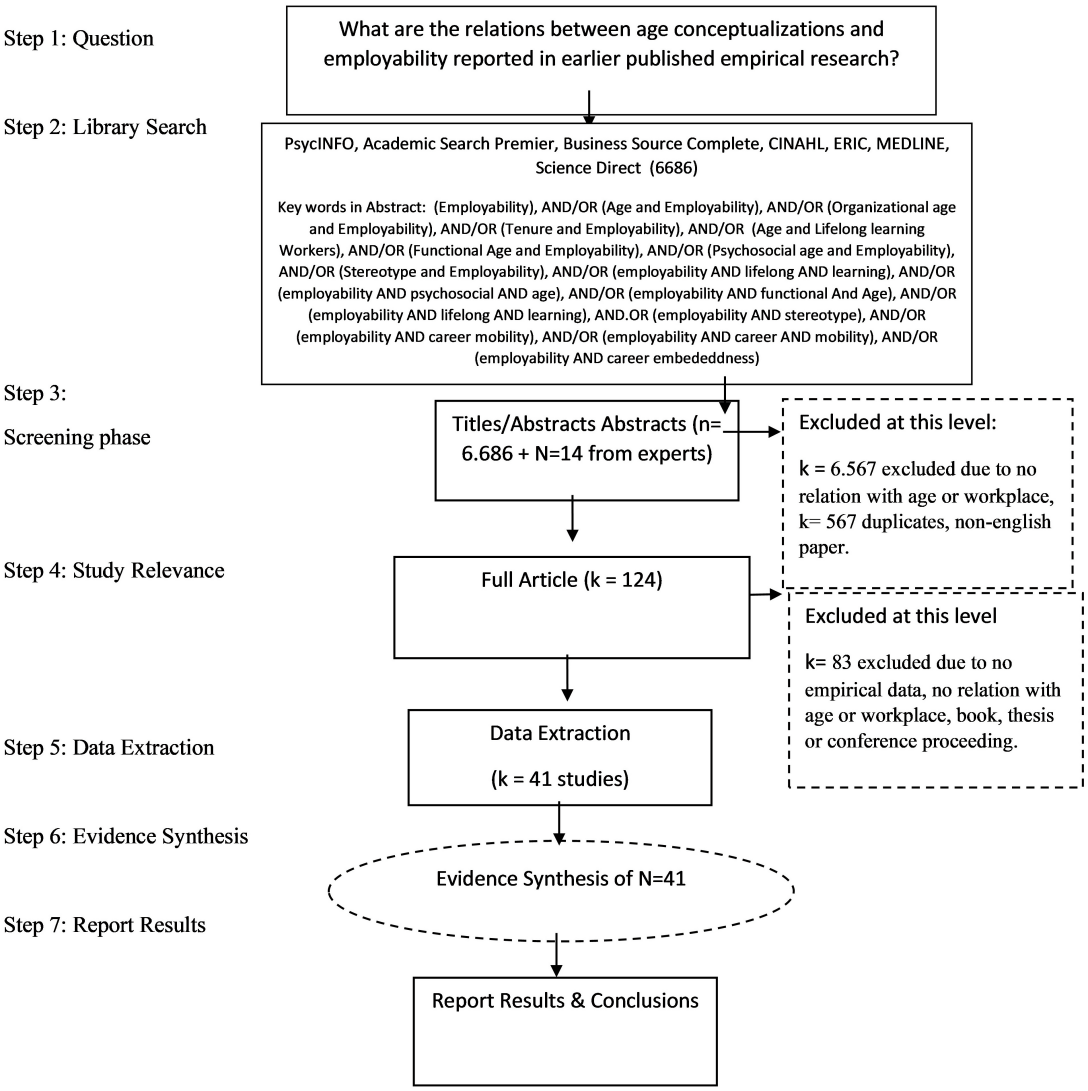
Flowchart of studies.

A systematic literature research was conducted using PsycINFO, Academic Search Premier, Business Source Complete, CINAHL, ERIC, MEDLINE, and Science Direct, and the search strings: (Employability), AND/OR (Age and Employability), AND/OR (Organizational age and Employability), AND/OR (Tenure and Employability), AND/OR (Age and Lifelong learning Workers), AND/OR (Functional Age and Employability), AND/OR (Psychosocial age and Employability), AND/OR (Stereotype and Employability), AND/OR (employability AND lifelong AND learning), AND/OR (employability AND psychosocial AND age), AND/OR (employability AND functional And Age), AND/OR (employability AND lifelong AND learning), AND.OR (employability AND stereotype), AND/OR (employability AND career mobility), AND/OR (employability AND career AND mobility), AND/OR (employability AND career embeddedness), which resulted in 6,686 hits (see Figure 1 for Flowchart).

### Selection of Studies

Study selection was conducted, independently, by the first author in pairs with the other co-authors of this paper, using the inclusion and exclusion criteria described below. Following systematic review guidelines (Petticrew and Roberts, 2006), we have drawn a flowchart to illustrate our selection of relevant empirical studies to be included in the systematic review (see Figure 1).

### Criteria for Inclusion of Studies

Selection based on title and abstract was performed by at least two reviewers (the first author and one of the other authors). Articles were excluded when both reviewers determined that the study did not meet the inclusion criteria. In case of disagreement or doubt, a third reviewer was consulted (cf. Steps 3–5 in Figure 1; resulting in an overall inter-rater agreement of 97.5%). More specifically, in order to be included, the title, abstract or method section were to include the topics age and employability; resulting in excluding 6,567 abstracts and 567 duplicates in Step 3 of our screening phase. Book chapters, dissertations, and conference proceedings were excluded. As a result, in Step 4 of the screening phase, *k* = 83 studies were excluded, as these articles did not include empirical data, or relevant variables, or referred to books, unpublished theses or conference proceedings.

Throughout the screening phases (cf. Steps 1–5 in Figure 1), the records were scrutinized using the following inclusion criteria:

- *Population:* Studies were included if the study population comprised at least *N* = 15 male or female workers (to ensure enough power for relationships to be studied) aged 21 years and older, that were active in the labor market.
- *Variables:* Studies were included if individual and/or supervisor ratings of employability and indicators of aging at work were measured, and included in the analyses.
- *Publication date:* Articles were selected if they were published between January 1980 and January 2019 (starting point of relevant studies and theory on the concept of employability).
- *Publication source:* Articles were selected if they were published in peer-reviewed journals.

Two raters evaluated the abstracts of the selected papers, and subsequently distinguished *k* = 41 relevant studies that met the inclusion criteria for the systematic review, and that could be used to answer Research Questions 1–5 (see Figure 1).

*The Standardized Index of Convergence (SIC) method*. As the number of studies that measure the same operationalizations of aging at work as well as of the concept of employability was too limited in number to perform a reliable meta-analysis, and as we wanted to avoid “vote counting” of the effects found in our systematic review, we decided to calculate the standardized index of convergence for the relationships found (see Research Question 5 and Tables 1, 2). According to Wielenga-Meijer et al. (2010), these SIC scores can be used to determine the degree of consistency in the effects found, when at least three studies can be used (and the SIC score does not require comparable effect sizes).

**Table 1:** Strength of the evidence for the relationships studied (Wielenga-Meijer et al., 2010).

**Sic value**					
**Number of studies**	**1.00 to 0.60**	**0.59 to 0.30**	**0.29 to−0.29**	**−0.30 to−0.59**	**−0.60 to−1.00**
1–2	Insufficient evidence (i.e.)				
3–5	++	+	0	–	——
≥6	+++	++	0	——	———

*0, inconsistent evidence; + (–), limited evidence for a positive (negative) relationship; ++ (——), moderately strong evidence for a positive (negative) relationship; +++ (———), strong evidence for a positive (negative) relationship*.

*Sic score is calculated by: n[positive]-n[negative]/n[total]*.

*The SIC ranges from −1 to 1. According to Wielenga-Meijer et al. (2010) values between 0.29 and −0.29 indicate that there is an inconsistent effect. Values between 0.30 and 1 indicate evidence for a positive relationship and values between −0.30 and −1 indicate evidence of a negative relationship. However, this does not give any information regarding the strength of the evidence. The strength of evidence is either “strong,” “moderate,” “weak,” or “inconsistent.” Strong evidence indicates that the findings are consistent across many studies (e.g., many studies find a negative or positive effect), whereas inconsistent evidence indicates that the findings*.

**Table 2:** Synthesis of results per type of age conceptualization and indicator of employability.

**Age conceptualization**	**Competence-based employability (*k* = 15 articles)**	**Labor market-based employability (*k* = 23 articles)**	**Combined competence-based and labor market-based measure of employability (*k* = 3 articles)**
**Chronological age (40 studies in total):**			
-Calendar age (*k* = 40 articles in total: 14 competence-based, 23 labor market-based, 3 combined)	0 **(inconsistent evidence)**	——— **(strong evidence negative)**	**-(moderately strong evidence negative)**
**Functional age (7 studies in total):**			
-Work ability (1 competence-based, 3 labor market-based, 2 combined)-Health (2 studies competence-based, 1 study labor market-based)	Insufficient evidence Insufficient evidence	**++** **(Moderately strong positive)** Insufficient evidence	Insufficient evidence Insufficient evidence
**Psychosocial age (2 studies in total):**			
-Stereotype (1 study: labor market-based perspective)-Time perspective (1 study: competence-based perspective)	Insufficient evidence Insufficient evidence	Insufficient evidence Insufficient evidence	Insufficient evidence Insufficient evidence
**Organizational age (13 studies in total):**			
-Job tenure (3 competence-based, 9 labor market-based, 1 combined measure)	—— **(moderately strong negative evidence)**	——— **(moderately strong negative evidence)**	Insufficient evidence
**Lifespan age (6 in total):**			
-Marital status (2 labor market-based)-Financial situation/salary (1 study competence-based)-Partner and children (2 study labor market-based; 1 competence based)	Insufficient evidence Insufficient evidence Insufficient evidence	Insufficient evidence Insufficient evidence Insufficient evidence	Insufficient evidence Insufficient evidence Insufficient evidence

The SIC score is calculated by means of n[positive]-n[negative]/n[total], and ranges from −1 to 1. Values between 0.29 and −0.29 indicate that there is an inconsistent effect, or in other words, that the results are mixed. Values between 0.30 and 1 indicate evidence for a positive relationship, and values between −0.30 and −1 indicate evidence of a negative relationship. However, this does not give any information regarding the strength of the evidence. We have distinguished between “strong,” “moderate,” “weak,” or “inconsistent” evidence (see Table 1). For instance, strong evidence indicates that the results are consistent across many studies (e.g., many studies find a negative or positive effect), whereas inconsistent evidence indicates that the results vary across the studies and no clear conclusion about the nature of the effect size(s) found can be established.

## Results

### What Are Relevant Descriptives of the Reviewed Studies (In Terms of Type of Research Population and Country)?

Our systematic review revealed *k* = 41 eligible studies that examined relationships between different conceptualizations of aging at work and employability. Although the reviewed studies included data from diverse countries (e.g., Central Europe, USA, Korea, Scandinavian countries), 53.7% (22 papers) were based on data collected among Dutch workers. Response rates, for those reported, ranges from 9 to 93%. Furthermore, the studies considered different blue-collar as well as white-collar jobs (different sectors and job types; low- and high qualified workers). The studies include a total of 46.826 individuals (*n* ranging from 119 to 6,696).

### What Are the Operationalizations of the Concept of Age Used in These Empirical Studies (e.g., Chronological, Functional, Psychosocial, Organizational, Lifespan Age)?

Considering the conceptualization of aging at work, most studies included calendar age [40 studies; 97.6%; total mean age of the respondents in the included studies was 41.68 years and the mean age range was 21–65 years old; Owuamalam and Zagefka (2014) included only psychosocial age in their study], whereas seven studies (17%; Lee et al., 2009; De Graaf et al., 2011; Van Vuuren et al., 2011; Nilsson and Ekberg, 2013) included indicators of functional age (like health and work ability), 13 studies included job tenure as an indicator of organizational age (31.7%; Kang and Kim, 2012; Van der Klink et al., 2014), six studies measured marital status or salary, partner, and children as indicator of lifespan age (14.6%; Ostroff and Clark, 2001; De Cuyper et al., 2008; De Graaf et al., 2011; Van der Klink et al., 2014; Peeters et al., 2016; Le Blanc et al., 2017), and two studies (4.9%) examined relationships between psychosocial age and employability (including remaining time and remaining opportunities; Froehlich et al., 2014).

### What Are the Operationalizations of the Concept of Employability Used in These Studies (e.g., Competence-Based vs. Labor Market-Based Measures or Both Types of Measures Included)?

The selected studies utilized diverse definitions and operationalizations of the concept of employability. Of the reviewed studies, 15 studies included a competence-based measure of employability (36.6%), and 23 studies included labor market-based measures (56.1%), whereas three studies measured both types of employability measures (Van Vuuren et al., 2011; Akkermans and Tims, 2017; Van Vuuren and Marcelissen, 2017; 7.3%). As a result, we will describe the significance of the results found for competence-based vs. labor market-based measures separately (see Research Question 5 and Table 2).

### Which Research Design Was Used (e.g., Cross-Sectional or Longitudinal Survey Research)?

Considering the research design, 34 studies were based on a cross-sectional design (82.9%) and 7 studies (17.1%) were based on a longitudinal panel study. This outcome indicates that only a few studies were able to tap more long-term developments in relationships between aging and employability.

### What Is the Direction and the Strength of Evidence of the Relationships Between the Different Age Operationalizations and Employability (e.g., Significant Positive, Negative, or Non-significant Effects Based on the SIC Method)?

*Competence-based measures*. Fifteen studies examined relationships between calendar age and competence-based measures (cf. Table 2). However, based on the SIC score inconsistent evidence was found across these studies. *Labor market-based measures*. More consistent findings were found for the relationships when labor market-based measures of employability were used. In particular, based on the SIC score, strong negative relationships were found between calendar age and labor market-based employability. Three studies, that included labor market-based measures, performed subgroup calendar age analyses by distinguishing between two age groups (i.e., employees of 40 years or younger vs. employees of 41 years or older; Van der Heijden et al., 2009; Sok et al., 2013) or between three age groups, that is, starters (20–34 years), middle-aged (35–49 years), and seniors (50 years or older) (Van der Heijden, 2002). Although the categorizations varied somewhat between these studies, all three found mostly negative associations of calendar age with employability. More specifically, Van der Heijden (2002), in her research on five dimensions of occupational expertise (Van der Heijden, 2000), being an important element of one's employability (Van der Heijde and Van der Heijden, 2006; Van der Heijden et al., 2018), found that for middle-aged employees, occupational skills and growth and flexibility were negatively related to employability, whereas for older employees, the extent of social recognition was (weakly) negatively associated with employability. For the latter group, the degree of growth and flexibility was (weakly) positively related to employability. This outcome is important in the light of nowadays' working life. In particular, Van der Heijden et al. (2009) found that supervisor ratings of employability were negatively related to overall promotions of the older employees, although self-reported employability was positively related to overall promotions for this group.

All in all, the studies that included competence-based as well as labor market-based measures found moderately strong negative relationships between calendar age and employability (revealing similar findings as the aforementioned findings of labor market-based measures).

Furthermore, Nauta et al. (2005) found that calendar age was positively related to productivity and employability in one's own job but negatively associated with employability in other jobs. Van Vuuren et al. (2011), in their cross-sectional survey among 178 school employees (mean age was 42.2 years), found a significant negative relationship between calendar age and labor market-based employability (Van Vuuren et al., 2011) as well as a positive relationship between *functional age* (measured as work ability) and employability. Finally, in their longitudinal survey study conducted among 284 low-qualified employees of 35 different companies, Raemdonck et al. (2008) found significant negative relationships of calendar age, on the one hand, and job mobility, vertical mobility, and job turnover, on the other hand, indicating that a higher calendar age was related to reduced job mobility, vertical mobility, as well as reduced job turnover.

With regard to *functional age* (i.e., health and work ability), five out of the seven studies that used this age operationalization found significantly positive associations of health with (aspects of) labor market-based measures of employability (Nielsen, 1999; Van Vuuren et al., 2011; Le Blanc et al., 2017; Van Vuuren and Marcelissen, 2017; Fleuren et al., 2018). According to the accompanying SIC score, this means that the relationship between functional age and labor market-based measures is moderately positive. The relationship between functional age and competence-based measures also tended to be positive, but the amount of studies was too limited and therefore resulted in a SIC score of insufficient evidence. Thus, most studies included in this review found that employees with high levels of (perceived) health and/or high work ability (perceptions) also reported higher labor market-based employability scores.

Thirteen studies operationalized age in terms of *organizational age* (i.e., job or organizational tenure). For organizational age, on the one hand, and competence-based as well as labor market-based measures of employability moderately negative relationships were found.

*Competence-based measures*. As regards the input- or competence-based measures of employability, Van Dam (2004), for example, found a significant relationship between job tenure and employability-related activities (like training) among 339 Dutch bank employees. This outcome indicates that employees with more experience at work were inclined to invest less time in employability-related activities. Lo Presti et al. (2018), using a sample of 254 Italian and 254 Finnish workers in small and medium-sized companies, only found significant negative relationships between job tenure and competence-based employability among the Finnish workers. Analogously, Van der Klink et al. (2014) found a significant negative relationship between job tenure and competence-based employability among a sample of 139 academic workers for the employability subscale anticipation and optimization of Van der Heijde and Van der Heijden (2006).

*Labor market-based measures*. Similar findings were found for the survey studies examining relationships between organizational age and labor market-based measures. For example, Berntson et al. (2006), in their cross-sectional study among a large sample of Swedish workers, found a negative relationship between job tenure and labor market-based employability. In line with this, De Cuyper et al. (2008) found a negative relationship between job tenure and labor market-based employability in a study using a sample of 559 Belgian workers. However, Kang and Kim (2012) found non-significant findings for the aforementioned relationship in their empirical work among 207 dyads of supervisors and their subordinates in Korea. Overall, the negative relationship between organizational age and labor market-based measures were large enough in number to generate a SIC score that indicated a moderately negative relationship.

*Psychosocial age, lifespan age and employability: inconclusive evidence*. Unfortunately, the studies examining relationships between psychosocial age as well as lifespan age, on the one hand, and employability measures, on the other hand, were either too limited in number or revealed mixed findings. Consequently, we have found insufficient evidence to draw strong conclusions regarding the direction and nature of these relationships. Nonetheless, we can describe some relevant results that we have found in our review study. For instance, Froehlich et al. (2016) found, in relation to the *psychosocial* operationalization of age (i.e., remaining opportunities and remaining time in one's occupational life), significant positive relationships between remaining opportunities, on the one hand, and the employability dimensions of anticipation and optimization as well as personal flexibility, on the other hand (indicators of competence-based employability; Van der Heijde and Van der Heijden, 2006). Only a negative significant relationship was found between the employability dimension occupational expertise (Van der Heijde and Van der Heijden, 2006) and remaining time. In other words, workers who perceived more opportunities to set new (learning-related) goals also reported higher anticipation and optimization as well as personal flexibility, while the perception of less remaining time was associated with lower levels of occupational expertise.

Six studies used a *lifespan* operationalization of age, and in five of these, significant effects were found [i.e., significant effects of financial situation, marital status, having children and salary; a non-significant effect was found in study of Peeters et al. (2016)]. In the study by De Graaf et al. (2011), employees' financial situation was positively related to the competence-based employability subscale of balance (Van der Heijde and Van der Heijden, 2006) rather than to the total score of employability. Van der Klink et al. (2014) found that marital status was negatively related to personal flexibility (being a subdimension in the operationalization of Van der Heijde and Van der Heijden, 2006) and that salary was positively associated with corporate sense (Van der Heijde and Van der Heijden, 2006). Furthermore, for the labor market-based measures, De Cuyper et al. (2008) also found a negative relationship between marital status and labor market-based employability, in their empirical work among a sample of 559 Belgian workers from different organizations. Whereas Ostroff and Clark (2001), in their study among a group of 545 workers, found that marital status was significantly related to more willingness to accept job change (any type of job change, and with or without relocating to other area; β = 0.09^*^), whereas having children was only significantly related to more willingness to accept job change when no relocation was needed. Finally, Le Blanc et al. (2017), in their research among a group of 180 Dutch workers, found that having a partner was related to higher employability.

*Longitudinal research*. Seven studies employed a longitudinal design (Berntson and Marklund, 2007; Berntson et al., 2008; Raemdonck et al., 2008; Biemann et al., 2012; Gerards et al., 2014; Akkermans and Tims, 2017; Fleuren et al., 2018). Two out of these longitudinal studies found no significant associations of calendar age with competence-based employability (Berntson et al., 2008; Akkermans and Tims, 2017; using lengths of time lags of 1 month and 1 year, respectively). The other five longitudinal studies found significant negative associations of calendar age with labor market-based employability (Biemann et al., 2012, using 20 years of employment and job mobility data of German workers; Berntson and Marklund, 2007, using a 1-year time lag among Swedish workers; Fleuren et al., 2018, using a 1-year time lag among Dutch workers; Raemdonck et al., 2008, using a 1-year time lag among Belgian workers) as well as a negative relationship between calendar age and competence-based employability, but also a positive relationship between functional age and competence-based employability (Gerards et al., 2014, using a 2-year time lag among Dutch workers). Overall, from our systematic review we can conclude that with aging workers reported lower rates of job mobility, turnover and vertical mobility, and that their self-perceptions about their competencies became lower.

## Discussion

The aim of this contribution was to present the outcomes of the, to the best of our knowledge, first systematic review of empirical research on the relationships between different age conceptualizations and indicators of employability. Our literature search resulted in 41 studies that met our inclusion criteria for the review. Of these studies, 15 studies were based on an input- or competence-based measure of employability, whereas 23 studies included an output- or labor market-based measure of employability, and three studies included a combination of both types of measures. Furthermore, most studies incorporated calendar age (40 studies, 97.6%) as an indicator of aging at work, and utilized a cross-sectional design (34 studies, 82.9 vs. 17.1% that used a longitudinal design) to collect their data.

Based on the significance of the Standardized Index of Convergence (SIC) method, different types of evidence were found for relationships between the age and employability measures. A great strength of having used this relative new SIC method (Wielenga-Meijer et al., 2010) for earlier research on age and employability measures is that it enabled us to better categorize the exact nature of the empirical evidence that was found pertaining to each of the distinguished types of relationships between a particular age conceptualization and indicator of employability that have been included in our review (i.e., from weak to strong, and from conclusive to inconclusive). For relationships between psychosocial age and lifespan age, on the one hand, and employability measures, on the other hand, too few studies were found, resulting in inconclusive evidence. Only for relationships between calendar age and labor market-based measures of employability, strong consistent negative relationships were found across the studies that were included in our review, and moderate positive relationships were found for functional age and labor market-based measures. However, for organizational age, on the one hand, and competence-based as well as labor market-based employability measures, on the other hand, only moderate negative relationships were found.

**Figure 2:**
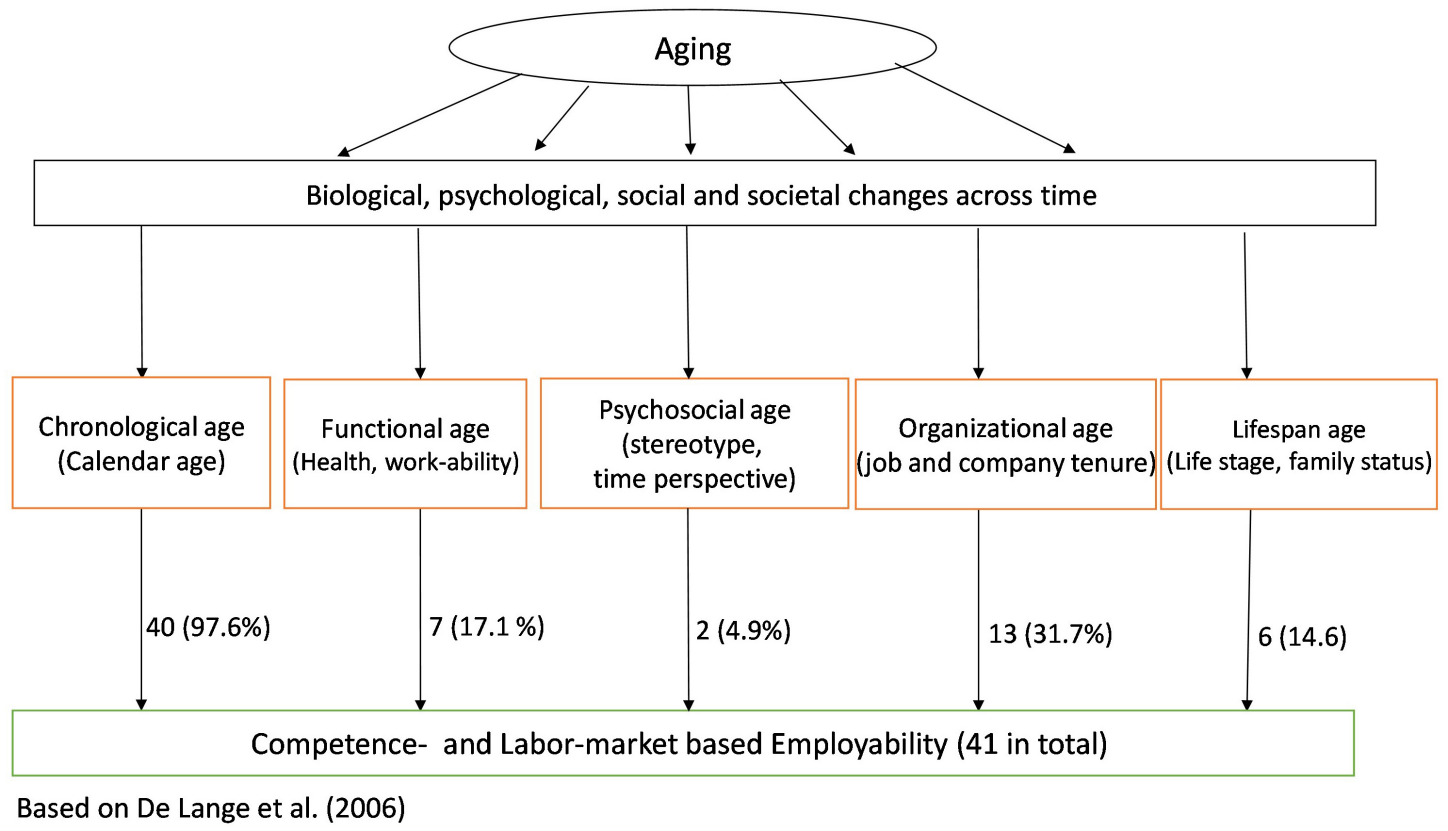
Number of studies per age conceptualization.

Overall, the results from our review revealed that the research on aging and employability can be portrayed as strong when examining relationships between calendar age and labor market-based measures, but that the scholarly work for the other types of relationships is still in its infancy, both in terms of the quantity as well as the methodological quality of the included studies. We base this rather strong conclusion on two conceptual and methodological concerns of the work presented in this review. First, we encountered a relatively small variety of conceptualizations of aging at work. In more detail, 40 studies included calendar age as an indicator of aging at work (including relatively younger to middle-aged and older workers with the mean age varying between 21 and 65 years) while other conceptualizations of aging (i.e., functional age, organizational age, lifespan age, and psychosocial age, see Figure 2) may be equally relevant in studying the associations of age with employability.

Second, we encountered a diversity in included definitions and operationalizations of the concept of employability, with empirical studies measuring the construct by using different types of scales for competence-based vs. labor market-based measures. We stress the need to measure employability at multiple levels (e.g., personal competencies as well as labor market opportunities). Studying employability at multiple levels and including structural or contextual factors provides a more comprehensive overview of the concept of employability and enables a better comparison of the results across studies, and in relation to age-related changes and processes across time.

Moreover, although the reviewed studies were performed in various countries (e.g., Europe, USA, Korea, Scandinavian countries), most of the included studies were based on Dutch and European data only, herewith restricting the generalizability of the findings across countries or making it possible for us to further address differences among these countries (see also future research agenda).

From a methodological point, we found that only seven studies out of our total amount of 41 reviewed studies employed a longitudinal design (17.1%), which seriously limits the conclusions regarding the nature and direction of the causality of the relationships found between aging and employability (De Lange et al., 2003). Aging employees may become either more or less employable across their lifespan, although the reversed hypothesis that highly employable employees feel more vital (i.e., psychosocial age) and healthier (i.e., functional age) compared to younger coworkers may be equally plausible. Moreover, in a one-lagged design or in a longitudinal design wherein the measurement points are too close to each other, one may find cohort effects instead of age effects, and therefore adopting such types of designs does not enable us to distinguish meaningful life course based developments from the entry of the labor market to the retirement or during bridge work in old age (Masche and van Dulmen, 2004; Zacher et al., 2018; Van der Heijden et al., 2020). Due to the limited data and different types of employability that have been used in previous scholarly work as well as regards the aging measures, we could not further disentangle age, time, or cohort effects in the reviewed study results. After all, reciprocal causal associations can only be examined in studies with multiple measurement points including the same type of measures. Therefore, future scholarly work in this field using more elegant research designs is urgently needed to more safely draw conclusions on the complex relationship between age and employability.

Furthermore, longitudinal studies are also needed to examine, for example, alternative mechanisms underlying the negative association between calendar age and labor market-based measures of employability found in this review. For example, the underlying mechanism or explanation can be more person or intrapersonal based. For example, employees with longer tenure may become transfixed in their jobs (i.e., immobile), which may lead to obsolete knowledge and skills (negative ability-related changes).

This review therefore presents a relatively dim prospect for older employees with regard to their labor market possibilities of obtaining and maintaining employment. However, as mentioned in the introduction section of this article, employability is not merely the result of the developmental (competency-increasing) efforts or dispositions of individual employees. We posit that these personal factors have to be backed up by structural or contextual factors at the level of one's job (e.g., work characteristics), at the level of the organization (e.g., career development support), and at a societal level (e.g., number of available jobs) levels (McQuaid and Lindsay, 2005). Since we both lack a substantial amount of longitudinal studies on aging employees, and research on the effect of employability-directed interventions at these four levels across time, we cannot simply conclude that older employees are less employable compared to their younger counterparts.

In sum using the outcomes of our systematic review, we were able to provide the first important synthesis of relationships between age conceptualizations and employability and, in doing so, to establish a sound or evidence-based ground for our conclusions that we will translate into recommendations for future research. In particular, the implications of our findings are that we call for new longitudinal complete panel research on aging and employability to:

- further examine the role of the “time”'factor, which is inherently attached to the concept of aging, but which is also affected by the changing labor market across time (Van der Heijden et al., 2020);
- examine the indistinct theoretical mechanisms underlying the associations of different conceptualizations of aging with competence-based and labor market-based employability; and
- consequently, examine the ambiguity regarding the causal association of aging with employability (e.g., are the two concepts reciprocally related across time?). More specifically, we call for new longitudinal complete panel studies with multiple time-points across time that would:

- include multiple conceptualizations of age (e.g., functional and psychosocial age);
- measure employability at multiple levels (e.g., with validated measures of personal competencies as well as internal and external labor market opportunities);
- examine these associations in different countries, and further disentangle whether differences in labor market dynamics across countries have an impact on the relationships;
- examine these relationships across various types of professions (e.g., both white- and blue-collar workers in the profit and non-profit sector);
- include senior age groups, like bridge workers aged 65 years and older;
- formulate more meaningful theory-based hypotheses on the relationships between aging and employability;
- combine both qualitative (e.g., narratives) and quantitative approaches (variable-centered and person-centered) in employability research.

For example, we call for more research aimed at further examining meaningful age-related life-events (such as marriage, parenthood, bereavement, and retirement) and employability-related choices and behavior (e.g., circumscription and compromise or career-related strategies and behavior of selection, optimization, and compensation across the lifespan (cf. Baltes et al., 1999; Gottfredson, 2002). Furthermore, Van Dam et al. (2016) proposed “age discrimination” as a possible contributing or theoretically meaningful factor that should be further examined in future scholarly work in relation to the decline in perceived employability (labor market position) as a function of calendar age, which we found in our selected research on labor market based-measures of employability (see also Van Harten et al., 2020).

To conclude, to the best of our knowledge, this is the first systematic review examining relationships between age conceptualizations and competence-based as well as labor market-based employability. Our review study shows a lack of systematic research on these topics and has resulted in an outline for a future research agenda. In addition, the empirical studies that have been performed in this domain so far are mostly cross-sectional in nature and focus primarily on calendar age as an indicator of aging. This limits the conclusions that we can draw from these studies' results. Besides the implications for future studies in this field, as mentioned above, we would therefore like to plead for a more fine-grained approach to input- and output-based types of measures for the concept of employability, herewith taking into account multiple age indicators and different types of employees' work context.

## Data Availability Statement

The original contributions presented in the study are included in the article/Supplementary Material, further inquiries can be directed to the corresponding author.

## Author Contributions

All authors listed have made a substantial, direct and intellectual contribution to the work, and approved it for publication.

## Conflict of Interest

TV was employed by a.s.r. Loyalis, Heerlen, Netherlands, and affiliated to the Open University, Heerlen, Netherlands. The remaining authors declare that the research was conducted in the absence of any commercial or financial relationships that could be construed as a potential conflict of interest.
