# Supplementary material for: Employable as We Age? A Systematic Review of Relationships Between Age Conceptualizations and Employability
Source: Front Psychol. 2021 Feb 5;11:605684. doi: 10.3389/fpsyg.2020.605684 (PMC7893083; doi:10.3389/fpsyg.2020.605684)
Supplement: Supplementary file 1 [file Table_1.docx]

Table 1: *Results of 41 Included Studies*

| **Authors** | **Population** | **Measures used: Competence-based and/or labor market-based** | | **Mean/SD** | | **Type of Age operationalization#** | | **Design (cross. or long.)** | | **RR (Response Rate)** | | **Results** | | **Aim of study** | |
| --- | --- | --- | --- | --- | --- | --- | --- | --- | --- | --- | --- | --- | --- | --- | --- |
| 1. Akkermans, J. & Tims, M. (2017)  **The Netherlands** | At Time 1, a total of 688 Dutch employees filled out the questionnaire. At Time 2, a total of 380 employees participated; The sample consisted of 109 females (59.6%) and 74 males (40.4%) | Competence-based as well as labor market-based:  Perceived employability was measured with eight items that reflect internal  and external employability (Akkermans et al., 2013a), which is based on De Cuyper and De Witte (2008). External employability was measured with four items, e.g. “I would find another job rather quickly if I searched for it” (a=5.83). Internal employability was also measured with four items, e.g. “In my current job, I am able to perform different types of tasks” (a=.88). Response categories ranged from 1 (completely disagree) to 5 (completely agree). Career competencies were measured with the 21-item Career Competencies Questionnaire (CCQ; Akkermans et al., 2013a) consisting of reflection on motivation (three items, e.g. “I can clearly see what my passions are in my work”, a=.68), reflection on qualities (four items, e.g. “I am aware of my talents in my work”, a=.80), networking (four items, e.g. “I am able to approach the right persons to help me with my career”, a=.78), self-profiling (three items, e.g. “I can clearly show others what my strengths are in my work”, a=.72), work exploration (three items, e.g. “I am able to explore my possibilities on the labor market”, a=.76), and career control (four items, e.g. “I can make clear career plans”, a=.87). Response categories ranged from 1 (completely disagree) to 5 (completely agree). | | Average age panel is of 24.81 years (Age range 16–30)  (SD tenure=1.81) in their current organisation. The total work experience was 4.82 years | | *Calendar age  *Organizational age (job tenure) | | Incomplete longitudinal panel study (independent on T1 and dependent on T2) | | Convenience sample: no baseline response. Attrition was: 55.2% | | **N.s.** Age as well as job tenure were not significantly related to career competencies and internal versus external employability (not significant; see Table 1 181); | | This study aimed to investigate whether career competencies could enhance an employees’ subjective career success in terms of perceived employability and work–home balance via job crafting behaviors. | |
| 2. Berglund, T. & Wallinder, Y. (2015)  **Sweden** | N = ranged between 1403 & 3031 employees of 21 countries aged 16-65 | Labormarket-based employability Employability theory (In the current article perceived employability  refers only to the perceived chances of transiting between jobs (Berglund  et al.  2014))  Employability:  “*How difficult or easy would it be for you to get a similar or better job with another employer if you had to leave your current job*?”  11-point scale ranging from 0 = Extremely difficult to  10 = Extremely easy.  Educational level is defined by the International Standard Classification of Education (ISCED). | | Age range 16-65 (no mean age or SD age available) | | *Calendar age: 16-29; 30-49; Range age 50-65. | | Cross-sectional and multi-level | | Between 47.1 & 74.7% | | **Sig neg**  Younger age groups report significantly higher employability compared to older age groups | | In an analysis of perceived opportunities among European employees, we argue that the concept of  ‘ employment security  ’ can lift the individualized  perspective on employability skills and attributes to a more structural and  institutional perspective. To enhance individuals  ’ chances of  finding a job,  educational and lifelong learning policies and active labor market  measures are believed to be important factors, besides general demand in  the labor market. Through multi-level modelling, we analyse the significance of individual and contextual factors on employees’ perception of their chances in the labor market. | |
| 3 Berntson, Marklund (2007)  **Sweden** | A total of 2536 individuals (84%) participated in the study at both time points and 1627 of those answered all items. It concerns a sample of the National Working Life Cohort from Sweden. | Labor market-based employability  In the present study, employability is seen as the individual’s perception of his or her  possibilities of getting new employment. The index included items related to the respondents’ perceived skills, experience, network, personal traits, and knowledge of the labor market (e.g., ‘‘I know of other organizations/companies where I could get new work’’). Respondents marked their agreement on a 5-point Likert scale. Employability was assessed at T1 with a Cronbach’s alpha of .88 | | Mean Age is 38.95 (SD= 7.13);  Emp T1 mean= 3.02 (SD=1.03) | | *Calendar age  * Functional age (Global health status, physical complaints, and mental well-being.)^[[1]](#footnote-1)^ | | 2-wave longitudinal study; Incomplete panel | | Total sample study comprised 1918 respondents. A missing value analysis indicated that our effective sample tended to comprise more women, older individuals, and more university educated individuals compared to those individuals that did not participate in the study | | **Sig neg**  -T1 Age-perceived employability sig neg (r=--.09 p<.05);  -T1 Global health-Emp sig pos (r=.10; p<.05)  -T1 Physical complaints-Emp sig neg (r= -.10;p<.05).  -T1 mental health-Emp sig pos (.23; p<.05)  Perceived employability was a significant predictor of two out of three outcome variables. Individuals with higher perceived employability had a tendency to report better health and well-being a year later | | The aim of the present study was to investigate the relationship between perceived employability and subsequent health, while controlling for baseline health, background factors, and work environment exposures | |
| 4 Berntson, Naswall, Sverke (2008)  **Sweden** | N=1730  Random, Swedish population,  ♀=52.8% | Calendar age  Labor market-based Employability,  6 items (Berntson & Marklund, 2007)  Generalized Self-efficacy, 8 items (Chen, Gully and Eden, 2001)  Education | | EmpT1= 2.98(1.03)  EmpT2= 3.02(1.03)  SE T1 = 3.54(0.70)  SE T2 = 3.56(0.70)  Age M=40.9 (7.1)  Range: 26-51yrs | | *Calendar age | | 2-wave Longitudinal  (1 year time-Lag)  SEM | | 80.5% | | **Sig neg**  Support for a four-factor model, e.g. distinct concepts,  Age T1 emp *r*=-.13 (p<.01)  Age T2 emp *r*=-.14 (p<.01) | | Analyze whether self-efficacy and employability are two distinct constructs and to investigate the direction of their relationship, if any. | |
| 5 Berntson, Sverke, Marklund (2006)  **Sweden** | Two national representative Swedish  samples are used, representing economic recession (1993, N= 4952) and prosperity (1999, N= 6696)). | Calendar age  Job tenure  Labor market-based employability  Perceived employability was measured by one item (‘How easy would it be for you to acquire new and comparable employment without moving?’). The response alternatives ranged from 1 (very hard) to 5 (very easy). | | Age (range: 16– 64 years)  Mean age sample 1 (1999)= 41.55 (SD=11.58);  Mean age sample 2 (1993): 40.08 (SD=12.03);  Job tenure sample 1:  M=12.80 Sd=10.61  Job tenure sample 2:  M= 12.26 Sd= 10.13  Emp sample 1:  M=2.82 Sd= 1.40  Emp sample 2  M=1.98 Sd=1.18 | | *Calendar age  *Organizational age (Job tenure) | | Cross-sectional; 2 samples | | response rates: 88 percent [1993] and 84 percent [1999]; second year the WES is attached to the LFS and carried out among those respondents in the LFS with employment, a total of about 15,000 individuals (response rates: 80.7 percent [1993], 66.9; no response rate analysis | | **Sig neg**  Age-empl sample 1:  R=-.28 (p<.05);  Sample 2:  R=.-.18 (p<.05)  **Sig neg**  Sample 1:  Job tenure-empl = -.17 (P<.05)  Sample 2:  Job tenure-empl =.-.18 (P<.05) | | The aim of the present study is to contribute to the understanding  of what predicts perceived employability. The identification of individual as well as contextual determinants of employability is facilitated by the application of two useful theories from the labor economic literature: human capital theory (e.g. Becker, 1993) and dual labor market theory (e.g. Doeringer and Piore, 1971). | |
| 6. Biemann, Zacher, Feldman (2012)  **Germany** | Data from a representative German sample (N=1259) | Labor market-based employability  Employment status retrospectively examined; no future-oriented employability measure. | | Mean age in 1984= 30.23 years, SD=7.84 | | *Calendar age | | 20 waves; longitudinal cohort study;  Human capital theory (Becker, 1975) suggests that the labor market rewards individuals' investments in their human capital  with higher wages and more job opportunities**.** | | Not described | | **Negative relation age and employment history:**  Here, we found that individuals who are younger were significantly more likely to have full-time, mobile career patterns or full-time to part-time career patterns; they were also less likely to be self-employed. Age and full time mobile career across 20 waves; indicating a negative relation age and labor market perspective employability | | Using 20 years of employment and job mobility data from a representative German sample (N=1259), we employ optimal matching analysis (OMA) to identify six career patterns which deviate from the traditional career path of long-term, full-time employment in one organization | |
| 7. De Cuyper, Bernhard-Oettel, Berntson, Witte, Alarco (2008)  **Belgium** | A sample of 559 respondents from divisions of seven Belgian organisations | Labor market-based employability was measured with four items which were developed by De Witte (2000). Respondents had to indicate their agreement with items such as “I am confident that I could quickly get a similar job” or “I am optimistic that I would find another job if I looked for one” (1 = strongly disagree; 5 = strongly agree). Reliability equaled .90 (N = 532, χ2 p < .05; GFI = .99; AGFI = .97; RMSEA = .09). | | Mean age was 34 years ( SD = 10.22). More women (64.7%; N = 355) than men (35.3%; N = 194) participated | | *Calendar age  *Organizational age (Job tenure)  *Lifespan age (marital status) | | Cross-sectional | | One industrial setting (N = 257; 46.0%) with a response rate of 87.6 per cent, and six smaller retail organisations ( N = 302; 54.0%) with response rates varying between 33 per cent and 58 per cent in five out of six organisations. The response rate was somewhat lower in one retail organisation (20.8%), probably because this organisation did not have a Human Resources department to actively support the research goals. | | **Age correlations not reported**  Sig neg job tenure-empl:  -.31 (p<.01)  Sig neg married-empl: r=--.13 (p.05) | | The current study’s aims are twofold: first, we investigate the relationship between employability and both work-related (engagement) and general (life satisfaction) well-being. Second, we study how employability may be relevant in times of high job insecurity | |
| 8. De Vos, Forrier, Van der Heijden, De Cuyper (2017)  **Belgium** | 2,137 professional workers; a Flemish career magazine targeting professional workers  51.3 percent were male | Competence-based Employability (Van der Heijde and Van der Heijden, 2006); perceived occupational expertise was measured using  six items developed by Van der Heijde and Van der Heijden (2006), which assesses employees’ beliefs in their capabilities and expertise (domain-related knowledge and skills and meta-cognitive skills) to adequately perform various tasks and to carry out the responsibilities of a job (e.g. “I consider myself competent to provide information on my work in a way that is comprehensible”). All items were scored on a five-point rating scale ranging from 1=“not at all,” to 5=“extremely” (Cronbach’s α in this study was 0.79). Perceived internal employability. Perceived internal employability was assessed with the  three-item scale developed by Eby et al. (2003). A sample item is: “There are many opportunities available for me in my company.” (Cronbach’s α=0.76). Perceived external employability. Perceived external employability was assessed using the  three-item scale developed by Eby et al. (2003). A sample item is: “I could easily obtain a comparable job with another employer.” All items were scored on a five-point rating scale, ranging from 1¼“strongly disagree” to 5¼“strongly agree” (Cronbach’s α=0.67). | | Their average age was 36 years (SD=9.05). We categorized respondents into three groups based on their calendar age: younger than 35 years (n¼ 1,003); between 35 and 49 years (n¼893); and 50 years or older (n¼241). | | *Calendar Age  Only as control variable:  *Organizational Age (job tenure and occupational position; but no results provided) | | Cross-sectional survey | | Conveniece sample; no response analysis | | No significant results for organizational age, and for subgroups age relations: Perceived occupational expertise related positively to perceived internal employability, and the relationships were similar in the subsample of younger (γ=0.50, po0.001), middle-aged (γ=0.48, p<0.001) and senior employees (γ= 0.47, po0.001). Likewise, perceived occupational expertise related positively to perceived external employability, and relationships did not differ significantly in strength across the three groups (γ=0.69, p<0.001 for younger, γ=0.68, p<0.001 for middle-aged, and γ=0.56, p<0.001 for senior employees). Perceived internal employability was negatively related to job search intensity and, consistent with H3, this relationship was significantly stronger among younger employees (γ=−0.33, p<0.001) in comparison with middle-aged (γ=−0.18, p<0.001) and senior (γ=−0.15, p<0.001) employees. Finally, perceived external employability related positively to job search intensity across all age groups (γ=0.19, p<0.001 for younger, γ=0.20, p<0.001 for middle-aged, and γ=0.19, p<0.001 for senior employees). Hence, H4 was not supported. | | The purpose of this paper is to advance our understandings of how employees’ occupational expertise is associated with job search intensity, through its assumed relationships with perceived internal and external employability in the internal and the external labor market. | |
| 9. Le Blanc, Van der Heijden, Van Vuuren (2017)  **The Netherlands** | N=180 employees from Dutch public service organizations; 67% male | Labor market-based employability  Opportunity to continue working was assessed by means of a  three-item scale on perceived internal and external employability by Verboon et al. (1999) and Veld et al. (2015) that were all scored on a five-point Likert scale ranging from: (1) absolutely not to (5) absolutely. An example item for external employability is: “I am confident that it is easy for me to find an attractive new job in a different organization.” Cronbach’s α was 0.79. | | Age ranged from 26 to 64 years. The mean calendar age was 48.99 years (SD = 8.42). | | *Calendar age,  *Organizational age (job and organizational tenure),  *Functional age (work ability reversed: low score is high WAI), and  *Lifespan age (partner and children). | | Cross-sectional survey | | 84% | | **Sig neg**  The opportunity to continue working was significantly negatively related to calendar age (β = −0.26; p < 0.01) and to functional age (β = −0.27; p < 0.01; reversed WAI score!), whereas it was significantly positively related to having a partner (β = 0.18; p < 0.05).  *organizational age (i.e., organizational tenure) had the strongest negative relationship with the opportunity to continue working. (r = −0.16; p <0.05)  **Sig. pos.**  *Moreover, functional age (work ability) was significantly negatively (reversed score; positive) related to opportunity to work (r = −0.29; p < 0.01)  * lifespan age appeared to enhance the perceived opportunity to continue working (in terms of having a partner:  *Calendar age was only important for the opportunity to continue working (r = −0.32; p < 0.01). | | sustainable employability is considered to be an important aspect of successful aging at work, this study used four different conceptualizations of aging at work to set up convergent and divergent validity of our operationalization of sustainable employability: calendar age, organizational age (job and organizational tenure), functional age (work ability), and lifespan age (partner and children). | |
| 10. Van Dam, K.(2004)  **The Netherlands** | There were 258 male and 81 female bank employees. | Competence-based Employability orientation was measured using seven items (see Appendix). Five items were taken from van Dam’s (2003a) sixteen-item scale measuring employees’ attitudes toward changes in the work situation; two items were developed that measured attitudes toward personal development. Cronbach’s alpha for this scale was .78. Six items were developed to measure employability activities (see Appendix). Cronbach’s alpha was .76 | | The average age of the participants was 40.4 years (SD=9.0); average organizational tenure was 14.6 years (SD=9.4). | | *Calendar age: only controlled for  *Organizational age (job tenure) | | Cross-sectional survey | | Three hundred and thirty nine (57%) of those contacted returned the questionnaire. | | **Negative relation job tenure** and employability orientation (r= -.36, p<.0001) and employability activities (r=-.35; p<..0001); **no results calendar age described.** | | To examine the Antecedents and consequences of employability orientation | |
| 11. Van Dam, K., van Vuuren, T. & Kemps (2017).  **The Netherlands** | The questionnaire was completed by 119 office employees (response 35%) of a Dutch public transport organization | Labormarket-based Employability was measured with Berntson et al.’s (2006) seven-item scale (α = .92) indicating one’s employability inside and outside the organization (e.g. ‘My skills are highly sought after at the labor market’ and ‘I could get another, similar job without much problems’) | | Mean age was 43.3 years (SD = 10.6); 32.8% were older than 50 years; 47.9% were male; mean tenure with the organization was 7.3 years (SD = 7.4).  Mean employability was: 3.19 (SD=.90) | | *Calendar age  *Organizational age (only controlled for job tenure; not reported results) | | Cross-sectional survey | | 35% response rate | | **Sig. Neg**  Negative relation between calendar age and employability:  R= -.25**) | | This study investigated the role of an intrinsically motivating job (intrinsic job value) and an age-supportive climate for three indicators of employees’ sustainable employment: | |
| 12. Fleuren, van Amelsvoort, de Grip, Zijlstra, Kant (2018) | Based on two-wave survey data from a sample of 2672 Dutch employees (ages 35‒65 years); (829 female, 1781 male, 62 missing) | Labormarket-based employability  3 single items; 1 self-constructed, 2 adapted from a self-report employability questionnaire by De Cuyper & De Witte (66), each measuring a different component. Labor market self-report:  "I am convinced that I could … (item 1: "…keep my current job until retirement…"; item 2: "…change jobs within my current organization..."; item 3: …get a job outside of my current organization…") … if I wanted to"; 5-point Likert | | Age range from 33.4‒65.0 (mean 52.9, SD 6.6) years remained for inclusion in the analyses | | *Calendar Age  *Functional Age (Work ability and health; but treated as outcome) | | Longitudinal 2-wave study | | 55.9% baseline response (4783 at baseline wave) | | **Sig. Neg.**  Age and Employability Time 1: r= -.12 (p<.01); Age Time 1 and Employability Time 2: r=-.10; p<.01)  **Sig. Pos.**  Work ability T1 and Employability T1: : r= .21 (p<.01);  Work ability T1 and Employability T12 : r= .14 (p<.01); | | Based on two-wave survey data from a sample of 2672 employees (ages 35‒65 years) multilevel regressions are estimated to analyze the effects of age and time on sustainable employability | |
| 13. Froehlich et al. (2014)  **Netherlands and Austria** | N = 780  Random, Dutch and Austrian workers:  4,153 employees were asked to participate. In total,  of which 780 (19 percent) were complete and used for further analyses: 613 of  the Dutch educational institution (16% response rate), 90 of the Austrian chamber, and 77 of the Austrian  IT company. The response rates of the latter two organizations (75 and 50 percent,  respectively)  ♀=59% | Calendar Age, Competence-based Employability (Van der Heijde and Van der Heijden, 2006);  -15 items occupational expertise (α=.91)  -8 items anticipation and optimization (α=.84)  -8 items personal flexibility (α =.77) | | Occupational expertise = 3.88 (0.6)  Anticipation and optimization= 3.27 (.75)  Personal flexibility=3.53 (.55)  Age M = 40.66 (SD= 11.19) | | *Calendar Age | | Cross-sectional | | Total 19%:  -16% of Dutch organization;  -75% of the Austrian chamber  -50% of the Austrian organization | | **Sig pos.**  Age + occupational expertise: r=.21**  Age + anticipation and optimization:  r=-.011**  Age + personal flexibility: r=.09* | | The purpose of this paper was to examine the effects of chronological age and formal  and informal learning activities on employability. Furthermore, indirect effects of age on employability  via learning activities were tested. | |
| 14. Froehlich et al. (2016)  **Netherlands**  **Austria** | Dutch emergency services organization in which the employees give first aid,  manipulate (complex) devices, and do administrative work; an Austrian IT  consultancy; and an Austrian consultancy  that offers a wider array of services. | Competence-based Employability: Three scales by Van der Heijde and Van der  Heijden (2006): occupational expertise (sample item: “I consider myself competent to  engage in in-depth, specialist discussions in my job domain;” α=0.92), anticipation and  optimization (sample item: “I take responsibility for maintaining my labor market  value;” α=0.78), and personal flexibility (Sample item: “I adapt to developments within  my organization;” α=0.73). | | Mean Occupational Expertise=4.16 (.53)  Mean Anticipation and Optimization= 3.58 (.61)  Mean Personal Flexibility= 3.68 (.51)  Mean Age: 41.85 (9.28) | | *Calendar age  *Psychosocial age: (future time perspective) | | Cross-sectional | | 282 (56 %) were complete and  used for further analyses. | | **No significant (Ns**) relations calendar age-employability (.04--.11)  -Ns: Remaining opportunities perspective-occupational expertise, r=-.05)  -remaining time-anticipation and optimization, r=.11  -remaining time and personal flexibility, r= .04  **Sig. pos:**  Remaining opportunities-anticipation and optimization, r=.30**  Remaining opportunities and personal flexibility, r=.34**  Remaining time and occupational expertise, r=-.15* | | The aim was to examine the relations among future time perspective, goal orientation, and employability.  Accordingly, we proposed that perceived remaining opportunities and  perceived remaining time at work relate positively to employability in terms of  occupational expertise, anticipation and optimization, and personal flexibility. | |
| 15. Gerards, De Grip, Witlox (2014)  **The Netherlands** | A randomly drawn sample of N=2500 regular employees of Philips Netherlands. | Calendar age, tenure  Utilization of courses offered through the voucher scheme: E-check course, self-insight training, employability advice, career pit-stop and route planner  Competence-based Employability awareness (5 items, 5-point Likert scales)  Personality traits (Locus of control, self-confidence, anxiety, self image, imagination, positive reciprocity, negative reciprocity, goal setting and career ambition; all scales >.70 alpha) | | Only measured at t0 (2008):  M(age)= 43.62; SD=9.30  M(tenure)= 17.04; SD=11.18 | | *Calendar age  *Organizational Age (tenure) | | Longitudinal (t1=2008, t2=2010) | | 38% (both surveys) | | Indirect significant effect: Job tenure in relation to voucher use and employability; no direct relations tested.  Workers with longer tenure spent their vouchers more often (odds ratio=1.10).  Calendar age was not significantly related to voucher use.  Voucher use was positively associated with a change (2010-2008) in employability awareness [e.g. “I myself am responsible for my future development (t2–t1)”, β=0.55, p<.001] | | This article studies the use and impact of a (‘Employability-miles’) voucher scheme, which could be used for participation in a restricted number of training courses aimed at stimulating employees to develop a more active attitude to their own employability | |
| 16. De Graaf, Peeters, Van der Heijden (2011)  **The Netherlands** | N=151 employees working at a Dutch Municipality; ♂=56%  ; White collar workers | Calendar Age, Competence-based Employability (5 scales: job expertise (15 items; 0.92 alpha); anticipation and optimization (7 items; 0.80); Personal flexibility (8 items; 0.79); Organizational flexibility (7 items; 0.82); Balance (8 items; 0.83; 1 item was exclude), 47-items in total **(Van der Heijde & Van der Heijden, 2006).**  -Intention to continue work (4 items; 0.92; Van Dam et al. (2009)  -Subjective Health | | Mean=52.6 (Sd= 4.8 years; Range:45-63years’  Job tenure (mean job tenure 20 years (Sd=10.6 years, but not examined in relation to employability); | | *Calendar Age,  *Functional Age measured as Health (general perceived health; 4 items; (α = 0.86; Adams en Beehr (1998).  *Lifespan age (financial situation)  *Job tenure: **no results provided** | | Cross-sectional (SEM analyses) | | **+/-**  151/650=  23,2% | | **N.s. calendar age**  **Sig lifespan age**: financial situation and subscale Balance (r=.18*)  **Sig. functional age**  employability and subjective health - R= .20** (relation balance and health)  R=.21** (relation personal flexibility and health)  -β=.22* for relation health and total mean employability score (mean of 5 subscales) | | To examine relations between employability and intention to continue work among older Dutch municipality workers.  Furthermore, the learning value and training opportunities were also related to further career management (not included in this table). | |
| 17. Kang & Kim (2012)  **Korea** | 207 supervisor-subordinate dyads in  Korean banking and financial institutions  300-paired surveys were distributed.  207 dyads remained  gender:  Education: 69.4 per cent with a bachelor’s degree or higher; | Calendar age, Organizational age: job tenure,  Labor market-based employability measured with a five-item scale based **on** Berntson  and Marklund (2007). The Cronbach’s alpha  (reliability measure) of this scale was 0.76. | | Age: M = 34.8 (range ¼ 23-47) years; work experience: M = 6.84; and  years with their current employer: M = 4.31. | | *Calendar age and *Organizational Age (job tenure) | | Cross-sectional | | Response rate of 76.3%for the employee group and 70.3% for the  supervisors | | **Sig. Calendar Age,**  **N.s. Organizational age**  Only correlations were reported; no regression analyses. on the results for employee level reported  **- Calendar Age and employability**  **(r= -.25**)**  **Job tenure and employability (r=**  **-.12; not significant)** | | This paper aimed to focus on a career perspective to investigate the association between  employee experience of job insecurity and work-related behaviors, specifically discretionary extra-role  and impression management behaviors. The second purpose was to analyze the interaction effect of  perceived employability and job insecurity on extra-role and impression management behaviors. | |
| 18. Kinnunen et al. (2011)  **Finland** | University teachers and researchers (n ¼ 1; 014) from two Finnish universities. Overall, 40  percent (n ¼ 408) were permanent employees, 49 percent (n ¼ 495) involuntary and 11 percent (n 111)  voluntary temporary employees.  ♀= 58%  Most respondents (54 percent) had education above a Master’s  degree, the average age was 43 years, and N = 58 % were women. | Calendar Age  Labor market-based employability  Perceived employability (PE) was measured using six items from the Employment Opportunity Index (Griffith  et al., 2005); e.g. “Given my qualifications and experience, getting a new job would not be very hard at all”) and networks (e.g. “I have contacts in other companies who might help me line up a new job”). The items of these two aspects loaded on the same factor in Confirmatory Factor Analysis (CFA): the loadings varied between 0.64 and 0.87 (see Table I). Responses were rated on a seven-point scale ranging from 1 (totally disagree) to 7 (totally agree). The items used resemble those items included in other PE measures (e.g., Berntson and Marklund, 2007; Rothwell and Arnold, 2007). | | Mean age= 43 years (SD = 11:1) | | *Calendar Age | | Cross-sectional | | **+/-**  Altogether 2,137 employees completed the questionnaire,  yielding a response rate of 47.4 percent | | **N.s**  **No significant relations** between Age and PE  Permanent workers:-r =0.09 (ns), involuntarily temporary workers: r =0.07 (ns)) | | The purpose of the present study was to examine how perceived employability relates to job  exhaustion, psychological symptoms, and self-rated job performance in involuntary and voluntary  temporary employees compared to permanent employees. | |
| 19. Nauta et al., (2005)  **The Netherlands** | N=1,463  Original sample was N=3,138, representative of the Dutch labor force. Only non-managerial employees were included in the study, which resulted in N=1,463.  ♀= 41.5%  40.6% were < 35 years, 29.2% were between 35 and 45 years, and 30.2% were >= 45 years.  35.3% had finished higher education, 42.3% average-level education, and 22.4% lower education. | ‘Proxies of employability measures, i.e.:  - Competence-based employability: Productivity and employability in one’s own job was measured with 4 items (α = .81), e.g.. ‘How often was the quality of your work worse than it actually should be?’ (reversed scored on a Likert Scale from 1-5).  - Labor market based Employability for other jobs was measured with 2 items (α = .84), e.g. ‘If I had to apply for a new job, I would soon find a good job.’ (Likert scale from 1-5) | | No means and SD’s were reported. | | *Calendar age | | Cross-sectional internet survey | | +/-  28.6% | | **Sig.** **positive relation between age and employability own function, but negative for other functions**  **Sig. pos**  Age was positively related to productivity and employability in one’s own job, both for employees with a specialist job (ß = .25**) and for employees with a generalist job (ß = .20**).  **Sig neg:**  Age was negatively related to employability for other jobs, and the relationship was stronger for specialist jobs (ß = -.32**) than for generalist jobs (ß = -.23**) | | Test whether employability declines faster with age for specialist jobs than for generalist jobs. | |
| 20. Nielsen, J. (1999)  **Denmark** | N= 5575 interviewed in 1995 | Labor market-based employability  Employability is the ability to become employed with the present skills and competencies (1 item: are you worried about not being able to get a new job with you present competencies and skills?) | | Not available | | *Calendar age  *Functional age (workability: ability to perform the work: how well the individual resources meet the job requirements: 1 item: is your workability reduced due to health problems?)) | | Cross-sectional survey | | RR is 80% | | 7% moderately reduced workability, 8% considerably reduced  **Sig. negative:**  strong association between age and negative thoughts on future employability  **Sig. positive**  Workability (reversed; positive result) are all sig. related to employability.  Age: odds ratio was 1.7 and 95% CI (1.4-2.0) for 35-44 years compared to 18-34 years and odd ratio 3.0 (26-3.5) for 45+ years compared to 18-34 years.  Workability: odds ratio was 1.4 and 95% CI (1.1-1.8) for moderate compared to not reduced and odd ratio 1,4 (1.2-1.8) for reduced compared to not reduced.  Gender: odds ratio was 1.5 and 95% CI (1.3-1.6) for female compared to male | | To examine the association between employability, workability and age | |
| 21. Nilsson & Ekberg (2013)  **Sweden** | N=191 whose **employment was terminated** because they had not been able to  Return to their regular work after taking a long-term sick-leave and rehabilitation measures | Competence-based employability  Individual employability was measured using a construct comprising seven items (DeFillippi & Arthur, 1994; Fugate, Kinicki, & Ashforth, 2004; McArdle, Waters, Briscoe, & Hall, 2007); Cronbach’s alpha was .90.  Generic health was measured using the validated standardized EuroQol (EQ-5D), which consisted of two parts. The instrument yielded a total of 243 possible health states with 1.0 indicating full health. The second part included the EQ visual analogue scale (EQ-VAS) with end points of zero for the worst imaginable health state and 100 for the best imaginable health state.  Work ability was measured using the validated Work Ability Index to evaluate the patients’ self-rated work ability (Ilmarinen, 1995). This instrument included 7 items. | | Mean Age= 50 years (No SD reported)  The respondents estimated their health as 6.22 (95% CI: 5.75-6.68) on a 10-pont scale.  The work ability of the cohort was estimated to be 5.15 (95% CI: 4.50-5.80) on a scale from one to ten.  The respondents estimated their employability to be 2.22 (95% CI: 2.00-2.45) on a 5-point rating scale. | | *Calendar Age  *Functional Age (health and work ability) | | Cross-sectional survey | | +/-  57% | | **N.s.** relations between calendar age and employability (r=.04)  Sig.  Positive relations for functional age: between employability and work ability (r=.26*); health (r=.36*); and education (r=.38*) | | To analyze how people who return to the labor market after long-term sickness absenteeism and subsequent job loss differ in employability, work ability, health, educational level, age, and gender compared to those who do not. | |
| 22. Lo Presti, Törnroos, Pluviano (2018)  **Italy and Finland** | Survey involving N=254 Italian and N=254 Finnish employees; participants were recruited among several small and medium organizations (SMOs). We adopted this sampling procedure because SMOs are the typical Italian organization (they employ the 79.8% ofworking population; ISTAT 2014) and in order to get the highest variability in response | Competence-based employability  Employability Orientation We used five items (e.g., I find it important to develop myself in a broad sense, so I will be able to perform different task activities or jobs within the organization^) from the original 7-item scale by Van Dam (2004). Responses were collected through a 4-point Likert scale (1 = completely disagree, 4 = completely agree). Cronbach’s alpha was .80 for Italy and .70 for Finland. | | Italy  Mean age was 38.68 years (SD 8.88); average organizational tenure was 11.17 years (SD 7.91).  Empl: Mean 1.82 (SD= 1.39)  Finland: Mean age was 45.26 years (SD 12); average organizational tenure was 15.66 years (SD 12.06)  Empl: Mean=1.94 (SD=1.25) | | *Calendar age (results not reported)  *Organizational age (job tenure) | | Cross-sectional survey (2 samples) | | Convenience sample; no response analysis | | Italy:  **N.s** tenure and employability (r=-.06; P>.05)  Age and employability: r= -.058 (ns)  **Finland:**  **Sig. negative** Tenure and employability (r= -.27; p<.01)  Significant negative:  Age and employability r= -.259 (p < .001) | | Aim was to investigate whether employability orientation acted as a mediator on the well-established relationship between self-esteem and psychological well-being | |
| 23. Peeters, Van de Ven, De Cuyper, Vlerick, De Witte (2014)  **Belgium** | N = 213 Belgian workers who voluntarily switched jobs; 72 (33.8%) male | | Labor Market-based employability  Scale of Berntson and Marklund (2007) (α = .80).5 items. I know other companies where I could get a job (response categories 1-5). | | Mean age= 36 (SD= 10.87)  Mean employability= 3.19 (SD= 0.77) | | *Calendar Age | | Cross-sectional | | No response rate analysis | | Negative relation between calendar age and employability: R=-.20 (P<.01) | | This study examines the motivational process of the Job Demands-Resources (JD-R) Model in a sample of recently dismissed (voluntary and involuntary) Flemish employees (N = 213). Specifically, the relations between three work-related resources (perceived employability, social support, and acceptance of feedback given during the exit conversation) and work-related behavior (in-role and extrarole behavior) were examined |
| 24. Peeters, De Cuyper De Witte (2016)  **Belgium** | 2 organizations: The selection resulted in a total sample size of 459 respondents. Age of the respondents ranged from 21 to 64 (M = 40.24y, SD = 10.50). 48.6% was female | | Labour-market based employability  Perceived employability was measured  with the four-item scale developed by De Cuyper and De Witte (2008). A sample item is: “I am optimistic that I will find another job  with another employer, if I looked for one” (1 = Strongly disagree; 5 = Strongly agree; Cronbach’s α = .94). | | *Calendar age;  Age of the respondents ranged from 21 to 64 (M = 40.24y, SD = 10.50).  Most participants had a partner (71.2%, N = 327) and 48.6% (N = 223) had children.  Tenure with the organization ranged from 1 to 43 years (M = 12.6, SD = 10.74). | | *Calendar age ;  Only control variable:  *Organizational age (Job tenure): not reported  *Lifespan age (partner, children): not reported | | Cross-sectional | | The invitation to the employees stressed that participation was voluntary. We received 354 out of 515 questionnaires distributed in one organization (response rate: 69%) and 288 out of 369 questionnaires distributed in the other organization (response rate: 78%) | | **Sig. Negative relation** Calendar age and employability:  R= -.44 (p<.01) | | To test curvilinear relationships between perceived employability and work engagement (vigor and dedication) and burnout (exhaustion and depersonalization) |
| 25. Ostroff, C. & Clark, M.A. (2001)  **USA** | N=545 employees of a Fortune 100 company  4 groups: 128 lateral move with career change  138 lateral move without career change  151 promotion with career change  128 promotion without career change  18% high school, 33% some college or 2 years degree, 26% college degree, 20% some graduate courses, 4% Ph.D  Only small significant difference between 4 groups on education level and tenure | Labor market-based Employability measured as willingnes**s** to accept a job change (LC: lateral move with career change, or LNC: lateral move without career change, PC: promotion with career change or PNC: promotion without career change : 1 item to indicate the chance from 0 to 100 if this did not require a move to another city or state and 1 item to indicate chance from 0 to 100 would accept if required to make a geographic relocation as well. | | Willing-no relocation M= 76.20 SD = 25.80  Willing-relocation  M=41.77 SD= 31.47  67% Male, 90% White, age: M= 41, SD= 9.7  Tenure: M= 14, SD = 8.9  85% Married/living with partner: M=1.15, SD=.36  Minor child: M=1.54 SD=.50 | | *Calender age  *Lifespan age: Marital status (1= married/living with someone, 2= single))  Children under age 15 (1=no, 2=yes)  *Organizational age: tenure in years | | Cross-sectional | | RR = 28% (565): | | **Sig. Pos**  Higher calendar age was significantly related to more willingness to accept job change and no relocation required for all four changes (β=.55**, and LC (β=.74**) and LNC (β=.81*) , but not for PC and PNC and also not for all and each job change if relocation was required.  **Sig positive**  Marital status was significantly related to more willingness to accept job change for all four changes (β=.09*),  Minor children status was only significantly related to more willingness to accept job change and condition no relocation for all four changes (β=.12**), and LNC (β=.37**).  **Sig pos:**  Tenure, was only significantly related to more willingness to accept job change and no relocation for all four changes (β=.51*) and LNC (β= 1.06*)  Interactions between age and gender were significantly related to more willingness to accept job change and no relocation for all four changes (β=-.63**), LC (β=-.91*) and LNC (β=-1.00*) and for relocation required for and LNC (β=-.77*).  Interactions between age and tenure were significantly related to more willingness to accept job change and no relocation for all four changes  (β= -.69**) and LNC (β= -1.37*) | | To examine relative importance of demographic and other factors in influencing employees willingness tot accept job changes | |
| 26 Owualmalam, C.K. & Zagefka, H. (2014)  **UK** | N=80 correlational study  N=56 experimental study | Labor market-based employability  3-items measure of Employability beliefs adapted of Bernstson and Marklund (2007): as a woman, I belief I could get a job in this society without problems, Women like me could easily find a job in this society, My identity als a woman makes it easy form e to get a job (1-6) | | No Calendar age statistics | | *Psychosocial age: (meta) stereotypes | | A cross-sectional correlational study and an experimental study | | Study 1: Eighty women were recruited at Keele University campus in the U.K. (Mage  ⫽ 20.63, SDage ⫽  2.14). At the time of study (2008/9), the ethnic composition of this university was 79.1% white and 20.9% ethnic minority (see Student Equality and Diversity Profile Report, 2010/2011); Study 2: ifty-six people of South Asian  ethnic decent were recruited from Staffordshire University, U.K. (32 men and 24 women; Mage=20.32, SDage= 1.39;  80.4% reported having British nationality). In terms of ethnic composition, 32.1% self-reported being Indian, 33.9% Pakistani, 24.2% Asian, 5.4% Bangladeshi, 3.6% Malay, and 1.8% Panthani. A between-groups design was used: | | **Negative relation:** Metastereotypes undermine employability beliefs because momentary self-doubts and particulary evident among members whose dispostional self-esteem is rather high. Study 1: R= -0.26 (p<..05). Study 2: beta= -0.30 (p<.05) for women versus ethnic minorities beta= -0.25 (p<.01) | | To investigate the effect of metastereotypes on employability beliefs of women and ethnic minorities | |
| 27. Raemdonck et al., (2008)  **Belgium** | N= 284 low-qualified employees working in 35 different Belgian companies  ♂=66% males | Calendar age;  Self-control or directedness concerning career steps and learning (14 items in total; Self directedness in learning was: α=.81 and self-directedness in career processes:α=.88)  **Labor market-based Employability** was measured by:  -Job stability, Job turnover, job mobility (categories: no mobility, horizontal, vertical in organization or external)  No alpha’s as these concerned categorical measures | | Mean age was 39 years (SD = 9.36), Mean job tenure: 20.48 years (SD = 10.31).  Job tenure was included, but not examined | | *Calendar age | | Longitudinal study (1-year follow-up study; through interview administered survey)  Incomplete panel design (see measures) | | +/- due to drop-out analyses  348 (82%) reported on both measures of the total N=408 of 35 different Belgian companies  Response selectivity was tested revealing significant effects between the response group and the dropout concerning the research variables | | **Significant negative relations between calendar age and employability**.  Significant relations were found between:  Age and no job mobility (r=.13*), age and no turnover; r=.16*) and age and vertical mobility (r= -.21**)  The odds ratio analyses also revealed significant effect for vertical mobility (β=-.15**); Age is related to reduced chances of vertical mobility. Furthermore, the effects of age on job turnover is also negative (β=-.26**) | | This study examines the relationship between self-directedness in  learning and in career and employability of low-qualified employees | |
| 28. Semeijn, Veld, Van Vuuren, Van Veen (2015)  **The Netherlands** | N=121 workers in elderly care (no information on gender) | Labor market-based employability  Self-perceived employability was measured using three items relating to the  employees’ labor market position (Verboon et al., 1999). All items were measured on  a five-point Likert-scale (ranging from totally disagree=1 to totally agree=5).  An example item was: “If I had to apply for another job, I would rapidly succeed in  finding one” (Cronbach’s α was 0.69). | | Mean age=44.72 SD= 9.88 years | | *Calendar age | | Cross-sectional survey | | 43% RR | | **Negative relation between age and employability:**  **r= -.23* (p<.05); β= -.02*** | | Aim was to examine relations between HR and work stress and control for age | |
| 29. Sok, Blomme & Tromp (2013)  **The Netherlands** | 2 samples: alumni hotel management school: Sample 1; 2006: 247 respondents: 88% Bachelor’s degree, 64% man, 12% Master’s degree.  68% < 40 or 40 years  Sample 2: only used for confirmatory factor analysis (2008) 135 respondents: 56% male, 88% Bachelor’s degree, 12% Master’s degree.  84,5% < 44 years | Competence-based employability  Ten Brink’s instruments (2004) were used to assess  employability. *Intra-organizational mobility intentions* were measured  with three items, for instance, “I am willing to perform my current position in another department”. Cronbach’s alpha .70  *Employee development*  was assessed by adding one item to Ten Brink’s instrument (2004):  “I develop new skills and knowledge for future jobs in the same profession”.  Cronbach’s alpha = .81  Labor market-based employability  Perceived labor market opportunities were measured  with three items, for instance: “If I wanted, I could easily find a  new job elsewhere”, as suggested but not researched by Ten Brink (2004).  Cronbach’s alpha = .75  Items of all measures were scored on a five-point scale,  ranging from 1 (totally disagree) to 5 (totally agree).  **Psychological contract** was measured with Ten Brink’s validated  questionnaire (2004) measuring the “state” of the contract,  one’s job), salary (e.g., competitive salary)  and intra-organizational mobility opportunities (e.g., opportunity  to work for another department). | | Sample 1,  our study sample (2006) consisted of 247 respondents: 157 males  (64%) and 90 females (36%). In this sample, the mean age was 37.3;  167 of the respondents were 40 years of age or younger (68%),  and 80 were over forty (32%).  Sample 2 (2008) consisted of 135 respondents:  75 males and 60 females (56% and 44%, respectively). In this sample,  most respondents were between 23 and 43 years old  (84.5%). | | *Calendar Age: younger workers < 40 or 40 years; older workers over 40 | | **Cross-sectional** | | +/-  No information about response rate included | | **Significant negative relation between calendar age and employability:**  older workers scored significantly  lower on all three employability measures: *Intra-organizational mobility intentions* (**T= 3.444*****), *Employee development*  (**T= 3.004****), *Perceived labor market opportunities* (**T= 2.628****)  For the  older employees (N=80), gender ((β=.21**), autonomy (β=.51***) and Job content (β=.30**) showed a positive relation with perceived labor market opportunities. Performance related pay (β=.40**) and promotion opportunities (β=.22*), relate positively with intra-organizational mobility intentions in group of older workers. Development  opportunities (β=.51***), showed positive relations with employee development in group of older workers, suggesting that these employer practices are important  for older employees, too. Such results are not congruent with other research (Van der Heijden et al., 2009; Van Dam, 2004). | | The aim was to assess the relationship between the psychological contract and self-perceived employability  (intra-organizational mobility intentions, employee development and perceived labor market  opportunities). | |
| 30. Stengard et al. (2015)  **Sweden** | N=129 Swedish workers  22% female  91% blue collar | Labor market-based employability  Employability 4-items (Berntson et al. 2006); Employability was measured with a four-item index (Berntson et al., 2006) reflecting the person’s subjective rating of their prospects of getting an equivalent or better job. An example item was ‘I could without problems get an equivalent job in another company/ organization’. Responses were measured on a five-point Likert scale ranging from 1 (to a very low degree) to 5 (to a very high degree). | | Mean age 43.1 (SD 10.9) | | *Calendar age  *Organizational tenure | | Cross-sectional survey | | 68.6% | | **Sig. Neg**  Correlation chronological age and employability r=-0,299 (p < 0,001).  Tenure correlated with employability -.28** | | To what extent perceptions of closure management and individual resources are associated with wellbeing and org. attitudes during plant closure | |
| 31. Van der Heijden (2002)  **The Netherlands** | N = 559 employees representative of the Dutch labor market (both profit and non-profit). | Competence-based employability  Professional expertise was measured with the multi-source (self-ratings and supervisor ratings) 78-item instrument comprising five dimensions (knowledge (17 items), meta-cognition (15 items), skill requirement (12 items), social recognition (15 items), growth and flexibility (19 items) by Van der Heijden (2000). All items refer to attributes or behaviors typically attributed to experts or outstanding performers in various fields.  Alpha self-ratings  (Cronbach’s alpha for all outcomes)  Knowledge: .83  Meta-cognition: .86  Skill req.: .84  Social rec: .83  Growth & Flex: .87  Alpha supervisor ratings  Knowledge: .93  Meta-cognition: .94  Skill req.: .94  Social rec: .94  Growth & Flex: .93  Labor market-based employability  To measure employability, 8 items were used with the following formulation: “What is the likelihood of transition to …?” The type of job transition on the dotted line being respectively: another job in the same domain as the employee’s present job, another job in another domain as the employee’s present job, a higher job in the employee’s own job domain, a higher job in the employee’s own organization or concern, a higher job outside the employee’s own organization or concern, an equivalent job in the employee’s own organizational unit, an equivalent job in the employee’s own organization or concern, an equivalent job outside the employee’s organization or concern. Cronbach’s alpha .68. | | Group means for employability:  18.89 (SD 2.70) for the starters (n = 113)  17.71 (SD = 3.72) for the middle-aged (n = 190);  13.80 (SD = 4.05) for the seniors (n = 103). | | *Calendar age  Age groups:  Starters (20-34 years)  Middle-aged (35-49 years)  Seniors (50+) | | Cross-sectional survey | | +/-  50% | | **Sig negative labor market:**  The relationship between age and employability was negative. The older the employee, the lower his or her employability (F = 61.38; *p* < .001).  The regression of employability on professional expertise and meta-cognitive knowledge was not significant.  When one of the other three dimensions of professional expertise was taken to be the predictor variable, interesting interaction effects between professional skills, social recognition, and growth potential on the one hand and age on the other hand on employability were observed.  Only in the case of the starters is the relationship between a particular dimension of professional expertise and the degree of employability positive. Among seniors, when the number of skills was the predictor, the relationship was negligible. When the predictor was the extent of social recognition, the relationship seemed to be weakly negative. When the degree of growth potential was the predictor, the relationship was weakly positive.  In the case of the middle-aged, the relationship was negative when skills or growth potential were the predictors, and negligible when the extent of social recognition was the predictor variable. | | The aim was to determine the relationship between five dimensions of professional expertise and the degree of future employability of higher level employees. | |
| 32. Van der Heijden, De Lange, Demerouti & Van der Heijde, (2009)  **The Netherlands** | 303 pairs of employees and supervisors working at a large Dutch company that produces building materials. | Competence-based Employability was measured with Van der Heijde and Van der Heijden’s (2006) instrument comprising five scales measuring: (1) occupational expertise (15 items); (2) anticipation and optimization (8 items); (3) personal flexibility (8 items); (4) corporate sense (7 items); and (5) balance (9 items). Examples are: “By virtue of my experience with him/her, I consider him/her … competent to be of practical assistance to colleagues with questions about the approach to work” (ranging from “not at all” to “extremely”) (*occupational expertise*), “(S)he is … focused on continuously developing him/herself” (ranging from “not at all” to “a considerable degree”) (*anticipation and optimization*), “(S)he adapts to developments within the organization …” (ranging from “very badly” to “very well”) (*personal flexibility*), “(S)he manages to exercise … influence within the organization” (ranging from “very little” to “a very great deal”) (*corporate sense*), and “The time (s)he spends on his/her work and career development on the one hand, and his/her personal development and relaxation on the other are … evenly balanced” (ranging from “not at all” to “a considerable degree”) (*balance*). The item sets for the employees and the supervisors were nominally identical and all measured on a six-point rating scale.  Objective career success was measured using three single items (Gattiker & Larwood, 1986). Objective hierarchical success was measured as the number of promotions. Number of promotions was defined as “any increase in hierarchical level and/or any significant increase in job responsibilities or job scope employees have experienced since joining their current organization” *(Organization-specific objective* ***hierarchical*** *success* [first item]) and in their entire career *(overall objective hierarchical success* [second item]). Objective financial success was measured, as *current gross income* (per month) [third item] | | Organization-specific promotions: Means and SD is 1.40 1.65  Overall promotions: Means and SD is 3.47 2.41  Income: Means and SD is 3429.81 3709.40 | | *Calendar age and differentiating between two age groups:  Youngsters: ≤ 40 years  Over-forties: > 40 years old. | | Cross-sectional | | RR= 91.8% | | **Sig pos. age and employability**  The significant structural paths showed that self-reported employability was positively related to overall promotions (*β* = .29, *p* < .001). Supervisor-rated employability was significantly yet negatively related to overall promotions (*β* = -.15, *p* < .05).  For the *younger employees (≤ 40 years)*, self-reported employability was significantly and positively related to both overall promotions and current gross income (*β* = .31, *p* < .001 and *β* = .35, *p* < .001, respectively).  The supervisor ratings of employability were positively related to current gross income (*β* = .19, *p* < .01).  The results for the *over-forties* were substantially different. For the older workers, we found that self-reported employability was positively related to overall promotions (*γ* = .33, *p* < .001) while supervisor ratings of employability were negatively related to overall promotions (*γ* = -.19, *p* < .05). All other structural relationships appeared to be non-significant. | | This study investigated whether the factor structure for self-reported versus supervisor-rated employability is similar across two age groups of workers. Second, we examined the predictive validity of employability in the light of objective career success using the two sources of raters. Finally, this study examined whether employee age moderates the relation between self- and supervisor ratings of employability on the one hand and objective career success on the other. | |
| 33. Van der Heijden et al. (2016)  **The Netherlands** | 330 pairs of employees  and supervisors (working at a large  Dutch company that produces building materials.  Employees working in a large variety of different types  of jobs at middle- and higher-level positions.  ♀=16.5% | Competence-based employability  five scales  measuring: (1) occupational expertise (15 items); (2) cor-  porate sense (seven items); (3) personal flexibility (eight  items); (4) anticipation and optimization (eight items); and  (5) balance (nine items).  All items were scored  on a six-point rating scale. Example items for the super-  visor ratings are: “By virtue of my experience with him/  her, I consider him/her … competent to be of practical  assistance to colleagues with questions about the approach  to work” (occupational expertise).  Alpha was .90 for  the self-ratings and .95 for the supervisor ratings. | | Mean age employee = 40.94 (9.20)  Mean occupational expertise= 4.77 (.42)  Mean corporate sense= 4.10 (.74)  Mean Personal flexibility= 4.52 (.42)  Mean Balance= 4.32 (.51) | | *Calendar age | | Cross-sectional | | Response rate 91.8% | | **Not Sig:**  Age - occupational expertise: r=.05  Age- corporate sense: r=.06  **Sig. negative:**  Age-Anticipation and optimization:  r= -.19**  Age-Balance self: r= -.16** | | The aim of this study was to test whether self- versus supervisor ratings of five  employability  dimensions (occupational expertise, corporate sense, personal flexibility, anticipation and optimization, and  balance) are associated with different learning characteristics in the workplace and whether age moderates these relation-  ships. | |
| 34.Van der Heijden et al. (2015)  **The Netherlands** | 9 Dutch primary schools in the eastern (more rural) part of the Netherlands. A total of 182 responses were received  (a response rate of 93.0 percent).  ♀= 75.8% | Labor market-based employability  Self-perceived employability was measured using three items relating to the  employees’ labor market position (Verboon et al., 1999). All items were measured on  a five-point Likert-scale (ranging from totally disagree=1 to totally agree=5).  An example item was: “If I had to apply for another job, I would rapidly succeed in  finding one” (Cronbach’s α was 0.76). | | Mean age= 42.3 (12.1 years)  Mean self-reported employability: 2.5 (0.8) | | *Calendar age | | Cross-sectional | | RR=93% | | **Not significant**  Age-Employability: r=-0.03 | | First aim of this study was to increase our knowledge of professional development  among teachers by focusing on the relationships between developmental opportunities at  work and positive work outcomes such as enhanced work engagement and self-perceived  employability. | |
| 35. Van der Klink, Van der Heijden, Boon, Williams van Rooij, (2014)  **The Netherlands** | N= 139 academic staff members  employed at the Dutch Open University in the Netherlands | Employee demographics: gender, age (measured as year of birth), and marital status (married/cohabiting or single)  Competence-based employability  Employability: the instrument initially developed by Van der Heijde and Van der Heijden (2006) was applied to measure employability, since this instrument  showed high levels of reliability in various settings (see Van der Heijden et al.,  2005). | | The average respondent’s age was 46 years (SD¼10.42), which is quite comparable to other universities in the Netherlands.  The Dutch Association of Universities, called VSNU, reported that, on average, about 30  percent of the academics working at Dutch universities is over 49 years old (VSNU, 2013).  The majority of the respondents was married (73 percent). Their average organizational  tenure was ten years (SD=6.06) | | *Calendar age, *Organizational age (job tenure), *lifespan age (marital status, salary) | | Cross-sectional study | | +/-  A total of 139 academics (74 men and 65 women) responded to an online survey,  yielding a 41.4 percent response rate | | **Sig. negative relations** were found between:  Age-anticipation (r= -.19*)  Age and Personal flexibility  (r= -.20*)  Marital status and personal flexibility (r= -.18*)  Job tenure and anticipation  (r= -.22**)  Age and Personal flexibility  (r= -.26**)  Salary and corporate sense  (r= 0. 26**)  Regression analyses only beta of salary and corporate sense remained significant (β=.20*) | | The purpose of this paper was  to explore the contribution of formal and informal learning to employability. | |
| 36. Van Vuuren, Caniels & Semeijn (2011)  **The Netherlands** | 178 employees from Dutch primary schools:  178 valid questionnaires The sample comprised of ♂=43 (24.2%) and ♀=135 employees (75.8%). The average size of the schools was 20.2 employees, of the employees worked 41.2% full-time. | Labor market- as well as competence-based Employability were measured using a 13 item scale by Verboon, De Feyter, & Smulders, (1999). The items refer to perceived chances on the internal and external labor market (3 items), employee willingness for mobility (7 items), and realized mobility (3 items). Example items are “I’m conﬁdent that it is easy for me to find an attractive new job in a different organization” and “I want to change my current job position for a different type of job”. All items were measured on a 5-point Likert-scale (absolutely not – absolutely). Cronbach’s α was .77. All items were measured on a 5-point Likert-scale (absolutely not – absolutely). Cronbach’s α was .77. **Workability** (Work Ability Index (WAI), Tuomi, Ilmarinen, Jahkola, Katajarinne, and Tulkki, 1994), All items were measured on 5-point Likert scales. of this scale was α = 0.78. Lifelong learning was divided into two groups; lower than average and higher than average. | | Calendar age:  average age of employees was 42.4 years  (SD = 12.1 ) . On average, the workers were employed 11.6 years (SD = 9.8 ) Relationship between Job tenure and age was not examined.  Employability M = 2.37 SD= 0.60  Work ability M = 40.92 SD= 4.48 | | *Calendar age: and *Functional age (Work ability) | | Cross-sectional | | 90,8% | | **Sig. negative relation calendar age and positive relation with functional age:**  R= -0.36, p < .01 between calendar age and employability  R= 0.18, p < .05 between functional age (work ability) and employability. For age, a statistically significant multivariate main effect was found, Wilks' Lambda = .835, F (6,340) = 5.350, p <.001. There appeared to be a significant univariate main effect for employability, F = 14.990, p <.001, but not for working ability or vitality. Finally, the interaction between age and lifelong learning on the dependent variables turned out to be significant, F (6,340) = 2.196, p <.05. The univariate tests showed that the interaction between age and life-long learning is significant for employability, F = 3.345, p <.005, and nearly significant for work ability F = 2.989, p <.10. | | The objective of this study was to examine in what way vitalizing HR practices influence employability of teachers. | |
| 37. Van Vuuren, T. & Marcelissen, F.(2017)  **The Netherlands** | N=866 (of 984) Dutch workers from 46 primary schools; 81% female teachers | Labor market- as well as competence-based employability  13-item measure of emloyability: employability is measured with 13 items (Verboon, De Feyter, & Smulders, 1999). Four subshales and one overall schale: 1: employee willingness for mobility to specialize . 2. employee willingness for mobility to change jobs . 3, perceived chances on the internal and external labor market (3 items and 4. realized mobility (3 items). mobiliteit; 5. employability total. Example items are “I’m conﬁdent that it is easy for me to find an attractive new job in a different organization” and “I want to change my current job position for a different type of job”. | | Mean age = 44 years (no SD score) | | *Calendar age  *Functional age (Work ability) | | Cross-sectional | | 87% response rate | | **Negative relation**  Age and employability r= -.28*** (p,.05**)**  **Positive relation** between work ability and employability: r=. 14*** | | To examine relations between age, gender and employability, vitality and work ability | |
| 38. Van Vuuren, van der Meeren, Semeijn (2013)  **The Netherlands** | N=263 (Total was 2974) Dutch workers from different profit and non-profit sectors; convenience sample; 58,9% male | 3 items measuring labor market-based employability (Verboon et al., 1999; Van Vuuren et al., 2011) Employability is measured as perceived chances on the internal and external labor market (3 items) (Verboon, De Feyter, & Smulders, 1999). | | The participants average age was 45.2  year. The youngest was 24 and the oldest participant 64. | | *Calendar age | | Cross-sectional | | 9% response baseline | | **Negative relation**  Age and employability  β=- .22*** | | To examine relations between HR practices and employability; age was a control variable | |
| 39. Van Vuuren, Semeijn, Caniels (2015)  **The Netherlands** | N= 1072 teachers from 61 different primary schools  17% was male | 3 items measuring labor market- based employability (Verboon et al., 1999; Van Vuuren et al., 2011). Employability is measured as perceived chances on the internal and external labor market (3 items) **(Verboon, De Feyter** | | Mean age= 43 years; SD= 11.1 years | | *Calendar age | | Cross-sectional | | 80% of total population | | Negative relation:  Age and employability  r= -. 22** (p<.01) | | To examine relations between HR practices and worker outcomes (including employability) for different age groups | |
| 40. Veld, Semeijn, Van Vuuren (2015)  **The Netherlands** | N=1.346 respondents from 91 primary school locations in the Netherlands | Labor market-based: Perceived employability (three items by Verboon et al., 1999). A sample item was: “I’m confident that it is easy for me to find an attractive new job in a different organization.” Five-point ratings scale (absolutely not-absolutely). Alpha is .75  Training, development opportunities (three items by Van Poppel and Kamphuis, 2004). A sample item was: “I get sufficient training opportunities”. Five-point scale ranging from strongly disagree – strongly agree). Alpha .83  Mobility supporting activities (three items by Van Poppel and Kamphuis, 2004). A sample item was: “My organization offers me the opportunity to conduct different tasks or to fulfill a different job.” Five-point scale ranging from strongly disagree – strongly agree). Alpha .77  Competence-based employability: Employee willingness for training and development (seven items by Van Vuuren et al., 2011). A sample item was: “I am willing to invest time in order to participate in training and development activities.” Five-point ratings scale (absolutely not – absolutely). Alpha is .89  Employee willingness for mobility (seven items from Van Vuuren et al., 2011). A sample item was: “I want to change my current job position for a different type of job.” Five-point rating scale (absolutely not – absolutely). Alpha is .76 | | Mean age=46.43; Sd= 11.21 | | *Calendar age | | Cross-sectional | | 81% | | **Age is negatively related** to Labor market-based perceived employability  r = -0.19**  Age and realized mobility: r = -0.19**  Age and mobility supporting activities:  r = -0.07*  Competence-based:  Age and Willingness for training and development  r = -0.29**  Age and Willingness for mobility r = -0.31** | | To investigate the moderating role of employees’ willingness to invest in training and development and willingness for mobility on the relationship between HR practices and employability. | |
| 41. Veld, Semeijn, Van Vuuren (2016)  **The Netherlands** | Data were collected in 2014 conducting a cross-sectional survey among managers (n=206) and employees (n=254) at a Dutch location of a large science-based multinationa | Labor market-based Employability can be defined as “an individual’s chance of a job in the internal and/or external labor market” (Forrier and Sels, 2003, p. 106).  Labor market-based Perceived employability was measured using a three item scale by Verboon et al.  (1999). The items refer to both the internal and external labor market. An example item for the external labor market is “I am confident that it is easy for me to find an attractive new job in a different organization.” All items were measured on a five-point Likert scale (absolutely not – absolutely). Cronbach’s α was 0.83. | | Mean age= 49.10, SD= 8.66 | | *Calendar age | | Cross-sectional | | In total 460 surveys were returned (33.3 percent overall response rate; 206 employees in a managerial position (34.9 percent) and 254 employees in a non-managerial position (32.2 percent) responded | | **Negative relation** between age and labor market-based perceived employability  -Age and perceived employability: r= -.42** | | The purpose of this paper is to examine three-way interactions among career control, career dialogue and managerial position in predicting perceived employability. The authors expected that participation in career dialogue strengthens the positive relationship between career control and employability. Furthermore, the authors expected that managers benefit more from career dialogue than employees | |

Note. : ♀= female; ♂= Male, * *p* < .05, ** *p* < .01, cross = cross-sectional, long.= longitudinal, #: Age operationalizations: calendar age, organizational age, lifespan age, functional age or psychosocial age, SEM= structural equation modeling; **N.s**.= not significant relations between age and employability, Sig = significant, +/-: response rate low or unknown (risk for biased sample selection)

Supplementary references of articles reviewed, but not cited in article

Berglund, T., & Wallinder, Y. (2015). Perceived Employability in Difficult Economic Times. *European Societies, 17*(5), 674-699. doi:10.1080/14616696.2015.1120879

Kinnunen, U., Mäkikangas, A., Siponen, S. M. K., & Nätti, J. (2011).Perceived employability. *Career Development International, 16*(2), 140 – 160. Retrieved from http://dx.doi.org/10.1108/13620431111115604

Peeters, E. R., De Ven, B. V., De Cuyper, N., Vlerick, P., & De Witte, H. (2014). The motivational process of the Job Demands-Resources Model among voluntary and involuntary dismissed employees during the term of notice. *Gedrag & Organisatie, 27*(2), 213-231.

Semeijn, J., Veld, M., van Vuuren, T., & van Veen, B. (2015). Duurzame inzetbaarheid in de ouderenzorg: HR-activiteiten als buffer bij werkstress? *Gedrag en organisatie, 28*(3). doi:10.5553/GenO/092150772015028003003

Van der Heijden, B. I. J. M., Van Vuuren, T. ., Kooij, T. A. M., & De Lange, A. H. (2015). Tailoring professional development for teachers in primary education. *Journal of Managerial Psychology, 30,*(1), 22 – 37.

Van der Heijden, B. I. J. M., Gorgievski, M. J., & De Lange, A. H. (2016). Learning at the workplace and sustainable employability: a multi-source model moderated by age. *European Journal of Work and Organizational Psychology, 25*(1), 13-30.

Van Vuuren, T., Semeijn, J. H., & Caniëls, M. (2015). Hebben oudere medewerkers op maat gesneden HR praktijken nodig? *Tijdschrift voor Toegepaste Arbowetenschap, 28*, 42-50.

Van Vuuren, T., Van der Meeren, W., & Semeijn, J. H. (2013). Vitaliteitsmanagement: HR activiteiten ter versterking van iemands duurzame inzetbaarheid. *Tijdschrift voor HRM, 12*, 7-27

Table 2: *Strength of the Evidence for the Relationships Studied (Wielenga-Meijer et al., 2010).*

| **Sic value** | | | | | |
| --- | --- | --- | --- | --- | --- |
| **Number of studies** | **1.00 to 0.60** | **0.59 to 0.30** | **0.29 to−0.29** | **−0.30 to−0.59** | **−0.60 to−1.00** |
| 1-2 | Insufficient evidence (i.e.) |  |  |  |  |
| 3-5 | ++ | + | 0 | - | -- |
| ≥ 6 | +++ | ++ | 0 | -- | --- |

: 0 = inconsistent evidence; + (-) = limited evidence for a positive (negative) relationship; ++ (--) = moderately strong evidence for a positive (negative) relationship; +++ (---) = strong evidence for a positive (negative) relationship.

Sic score is calculated by: n[positive]-n[negative]/n[total]

The SIC ranges from −1 to 1. According to Wielenga-Meijer et al.(2010) values between 0.29 and −0.29 indicate that there is an inconsistent eﬀect. Values between 0.30 and 1 indicate evidence for a positive relationship and values between −0.30 and −1 indicate evidence of a negative relationship. However, this does not give any information regarding the strength of the evidence. The strength of evidence is either ‘strong’, ‘moderate’, ‘weak’ or ‘inconsistent’. Strong evidence indicates that the ﬁndings are consistent across many studies (e.g. many studies ﬁnd a negative or positive eﬀect), whereas inconsistent evidence indicates that the ﬁndings

Table 3: *Synthesis of Results per Type of Age Conceptualization and Indicator of Employability*

| Age conceptualization | Competence-based employability (k = 15 Articles) | Labor market-based employability (k = 23 Articles) | Combined Competence-based and labor market-based measure of employability (k = 3 Articles) |
| --- | --- | --- | --- |
| *Chronological age (40 studies in total):  -Calendar age (k= 40 articles in total: 14 competence-based, 23 labor market-based, 3 combined) | 0 **(inconsistent evidence)** | **--- (strong evidence negative)** | - **(moderately strong evidence negative)** |
| *Functional age (7 studies in total):  -Work ability (1 competence-based, 3 labor market-based, 2 combined)  -Health (2 studies competence-based, 1 study labor market-based) | Insufficient evidence  Insufficient evidence | **++ (Moderately strong positive)**  Insufficient evidence | Insufficient evidence  Insufficient evidence |
| *Psychosocial age (2 studies in total):  -Stereotype (1 study: labor market-based perspective)  -Time perspective (1 study: Competence-based perspective) | Insufficient evidence  Insufficient evidence | Insufficient evidence  Insufficient evidence | Insufficient evidence  Insufficient evidence |
| *Organizational age (13 studies in total):  -Job tenure (3 competence-based, 9 labor market-based, 1 combined measure) | --**(moderately strong negative evidence)** | **--- (moderately strong negative evidence)** | Insufficient evidence |
| *Lifespan age (6 in total):  -Marital status (2 labor market-based)  -Financial situation/salary (1 study competence-based)  -Partner and children (2 study labor market-based; 1 competence based) | Insufficient evidence  Insufficient evidence  Insufficient evidence | Insufficient evidence  Insufficient evidence  Insufficient evidence | Insufficient evidence  Insufficient evidence  Insufficient evidence |

1. the present study included three different indicators of health and well-being: global health, physical complaints, and mental well-being. The three indicators were all assessed at both T1 and T2. Health at T2 was used as the dependent variable while health at the baseline was controlled for. First, a single question was utilized to measure the global health status of the respondents (Statistics Sweden, 2002). The respondents answered

   on a 5-point scale (very bad, bad, not bad or good, rather good, very good) to the item ‘‘How do you perceive your health status at the moment?’’ Second, physical complaints was measured using a 5-item mean value index (Statistics Sweden, 2003), which covered lower and upper back pain, as well as pain in the shoulders, hips, and wrists. Participants answered on a 5-point scale (not at all/seldom in the latest three months, a couple of days a month, one day a week, a couple of days a week, every day) addressing how often they experienced the symptoms. Cronbach’s alpha for the physical complaints index was .83. Third, an index of 10 items was used to measure mental well-being (Bech, Gudex, & Staehr Johansen, 1996). Respondents indicated on a 4-point scale (never, sometimes, often, all the time) how often they had felt any of the 10 symptoms during the last week (e.g., ‘‘I feel calm and peaceful,’’ ‘‘I have felt adjusted to my life situation’’). [↑](#footnote-ref-1)
